# Systematic review of Buzhong Yiqi method in alleviating cancer-related fatigue: a meta-analysis and exploratory network pharmacology approach

**DOI:** 10.3389/fphar.2024.1451773

**Published:** 2024-11-05

**Authors:** Ji Zeng, Qi Wu, Xu-Dong Meng, Jian Wang

**Affiliations:** ^1^ Clinical Pharmacy Department, Department of Pharmacy, Ma’anshan City Hospital of Traditional Chinese Medicine, Ma’anshan, Anhui, China; ^2^ Inflammation and Immune Mediated Diseases Laboratory of Anhui Province, Anhui Institute of Innovative Drugs, School of Pharmacy, Anhui Medical University, Hefei, Anhui, China; ^3^ Department of Pharmacy, Ma’anshan City People’s Hospital, Ma’anshan, China; ^4^ The Thyroid and Breast Surgery Department, Ma’anshan City People’s Hospital, Ma’anshan, China

**Keywords:** Buzhong Yiqi, cancer-related fatigue, meta-analysis, mechanism, network pharmacology

## Abstract

**Objectives:**

Cancer-related fatigue (CRF) is a prevalent and distressing symptom experienced by many cancer patients, necessitating effective treatments. This study utilizes meta-analysis and network pharmacology to comprehensively assess the efficacy of the Buzhong Yiqi prescription in alleviating cancer-related fatigue and to preliminarily explore the mechanism of its core drugs.

**Methods:**

We included randomized controlled trials (RCTs) in cancer patients. The inclusion criteria encompassed a diagnosis of cancer-related fatigue, without limitation on cancer type, the experimental group receiving Buzhong Yiqi prescription, the control group receiving conventional treatment, patients awaiting treatment, and articles published in either English or Chinese. We conducted a search through 29 February 2024, across PubMed, Cochrane Database of Systematic Reviews, Cochrane Controlled Clinical Trials (CENTRAL), China Biomedical Literature Service (CBM), China National Knowledge Infrastructure (CNKI), WANFANG Database, and Weipu Database (VIP). Journal articles that met the inclusion criteria were selected for inclusion. Two independent investigators evaluated the quality of the included studies. A meta-analysis was performed utilizing the Stata 12.0 software package, where estimates of cancer-related fatigue were aggregated through the application of a random-effects model. We employed the Cochrane Risk of Bias Tool to evaluate potential biases in RCTs. The primary outcome measures utilized to assess the efficacy and safety of CRF treatment comprised the Revised Piper Fatigue Scale (PFS-R) and the Quality of Life Questionnaire Core 30 (EORTC QLQ-C30). The secondary outcomes encompassed the KPS score, the effective rate, the TCM syndrome score, and an evaluation of adverse reactions. The Traditional Chinese Medicine Systems Pharmacology (TCMSP) was utilized to identify the active ingredients and targets of BZD. Additionally, the Drug bank, Therapeutic Target Database (TTD), DiaGeNET, and GeneCards databases were utilized to retrieve relevant targets for CRC. The Venn diagram was employed to identify overlapping targets. Cytoscape software was utilized to construct a network of “herb-ingredient-target” and identify core targets. GO and KEGG pathway enrichment analyses were performed using R language software.

**Results:**

In comparison to the control group, patients with CRF who received BZYQ prescription exhibited marked improvements in KPS score, QLQ-C30 quality of life score, and effective rate. Conversely, PFS, TCM syndrome score, and adverse reaction assessments significantly decreased. The primary active ingredients in its core drugs may exert a positive therapeutic effect on CRF by targeting molecules such as AKT1, IL6, IL1B, PTGS2, CASP3, ESR1, and BCL2, as well as through signaling pathways including TNF, IL17, TLR, NF-κB, and C-type lectin receptor.

**Conclusion:**

BZYQ demonstrates significant efficacy in treating CRF with minimal adverse reactions. It can serve as a fundamental treatment for CRF in clinical practice, and the medication can be tailored to individual patients for personalized therapy. The potential pharmacological mechanism of BZYQ in treating CRF, as predicted by network pharmacology, offers a molecular foundation for clinical CRF treatment.

**Systematic Review Registration:**

https://inplasy.com, identifier INPLASY202430025

## Introduction

Cancer patients suffer from a complex range of symptoms, including pain, weakness, sleep disturbances, and fatigue, throughout their treatment (Lawrence et al., 2004). These symptoms may limit the ability to function in activities of daily living. Fatigue is a particularly common and troubling symptom for cancer patients that has a negative impact on quality of life (QoL) throughout all phases of treatment and stages of the illness (Bower et al., 2000; Curt et al., 2000). Cancer and its treatments often trigger fatigue, either directly or indirectly through associated toxicities. The majority of cancer patients experience CRF during active treatment, with peak fatigue occurring at the end of treatment and gradually decreasing thereafter (Groenvold et al., 2007; Braun et al., 2008). A significant proportion of cancer survivors who are disease-free experience fatigue for years after completing active treatment (Braun et al., 2008). CRF is a complex and multidimensional condition encompassing environmental, physical, affective, cognitive, psychosocial, and spiritual factors (Bower, 2014). Its rapid onset, severe symptoms, persistence, and unpredictability significantly diminish the QoL and survival of cancer patients (Vogelzang et al., 1997). Despite the growing number of studies on the mechanism, treatment, and care of CRF, western medicine still lacks comprehensive evaluation and effective treatment for CRF (Thong et al., 2020). The main treatment drugs for CRF are central nervous system stimulants and corticosteroids, but their limited effects and obvious adverse reactions make them difficult to promote in clinical practice (Bower, 2014; Thong et al., 2020).

Traditional Chinese medicine (TCM) posits that CRF falls under the category of “fatigue,” and the symptoms of fatigue discussed in the “Golden Essentials” closely resemble the clinical manifestations of CRF (Berger et al., 2015). Currently, TCM prescriptions are being explored as a new approach to treating CRF (Hsu et al., 2012). The Buzhong Yiqi (BZYQ) prescription has been validated to have a certain therapeutic effect on CRF, with its core therapeutic drugs including: Astragalus (huangqi), Ginseng (renshen), Atractylodes Rhizoma (baizhu), Licorice (gancao), Tangerine peel (chenpi), Angelica sinensis (danggiu), Ascending hoist (shengma) and Bupleurum (chaihu). Depending on the specific conditions of individual patients, the basic medication is adjusted accordingly (Berger et al., 2015).

Recently, network pharmacology has utilized systems biology theory to analyzed biological system networks, select specific signal nodes for designing multi-target drug molecules, emphasize multi-channel regulation of signaling pathways, improve drug treatment efficacy, and reduce toxic side effects (Jiashuo et al., 2022). This aims to enhance the success rate of new drug clinical trials and reduce the cost of drug research and development (Jiashuo et al., 2022; Nogales et al., 2022).

Our study involved systematic meta-analysis to assess the efficacy and safety of BZYQ in treating CRF. In accordance with pertinent clinical guidelines, the PFS-R and the EORTC QLQ-C30 were chosen as primary outcome measures to assess the effectiveness and safety of CRF therapy. The secondary outcomes were KPS score, effective rate, TCM syndrome score, and assessment of adverse reactions. Additionally, we utilized network pharmacology to explore the pharmacological mechanism of BZYQ in CRF treatment, aiming to lay the groundwork for further investigation into the mechanisms of TCM ingredients in CRF treatment.

## Materials and methods

The protocol was registered on the International Prospective Register of Systematic Reviews (INPLASY, https://inplasy.com; registration No. INPLASY202430025) on 7 March 2024.

### Search strategy

We searched the digital databased PubMed, Cochrane Database of Systematic Reviews, Cochrane Controlled Clinical Trials (CENTRAL), China Biomedical Literature Service (CBM), China National Knowledge Infrastructure (CNKI), WANFANG Database, and VIPP Database from there inception to 29 February 2024. A search strategy was devised for each database (Table 1 in the Supplementary Figure S1), targeting articles published in English or Chinese.

### Inclusion and exclusion criteria

For inclusion, studies had to meet the specified criteria: 1) Utilization of a randomized controlled trial (RCT) design, 2) Inclusion of adult participants (≥18 years) with cancer, 3) Measurement of cancer-related fatigue (CRF) severity as an outcome, with primary outcomes including progression-free survival rate (PFS-R) and EORTC QLQ-C30, and secondary outcomes encompassing KPS score, effective rate, TCM syndrome score, and assessment of adverse reactions, 4) Evaluation of CRF severity not limited to its status as an adverse effect of cancer treatment, 5) Utilization of the BZYQ prescription as an intervention, 6) Avoidance of using reduced energy, vitality, or vigor as measures of fatigue, as these constructs are qualitatively distinct from CRF.

We excluded studies using BZYQ prescription as a control group and those not primarily focused on combination therapy. In the context of our study, while CRF may have been measured in studies utilizing BZYQ as a comparator, our primary aim was to ascertain whether BZYQ prescription therapy surpassed conventional treatments, including methylphenidate and corticosteroids. Consequently, we excluded articles that employed BZYQ as a control group.

### Study selection

**FIGURE 1:**
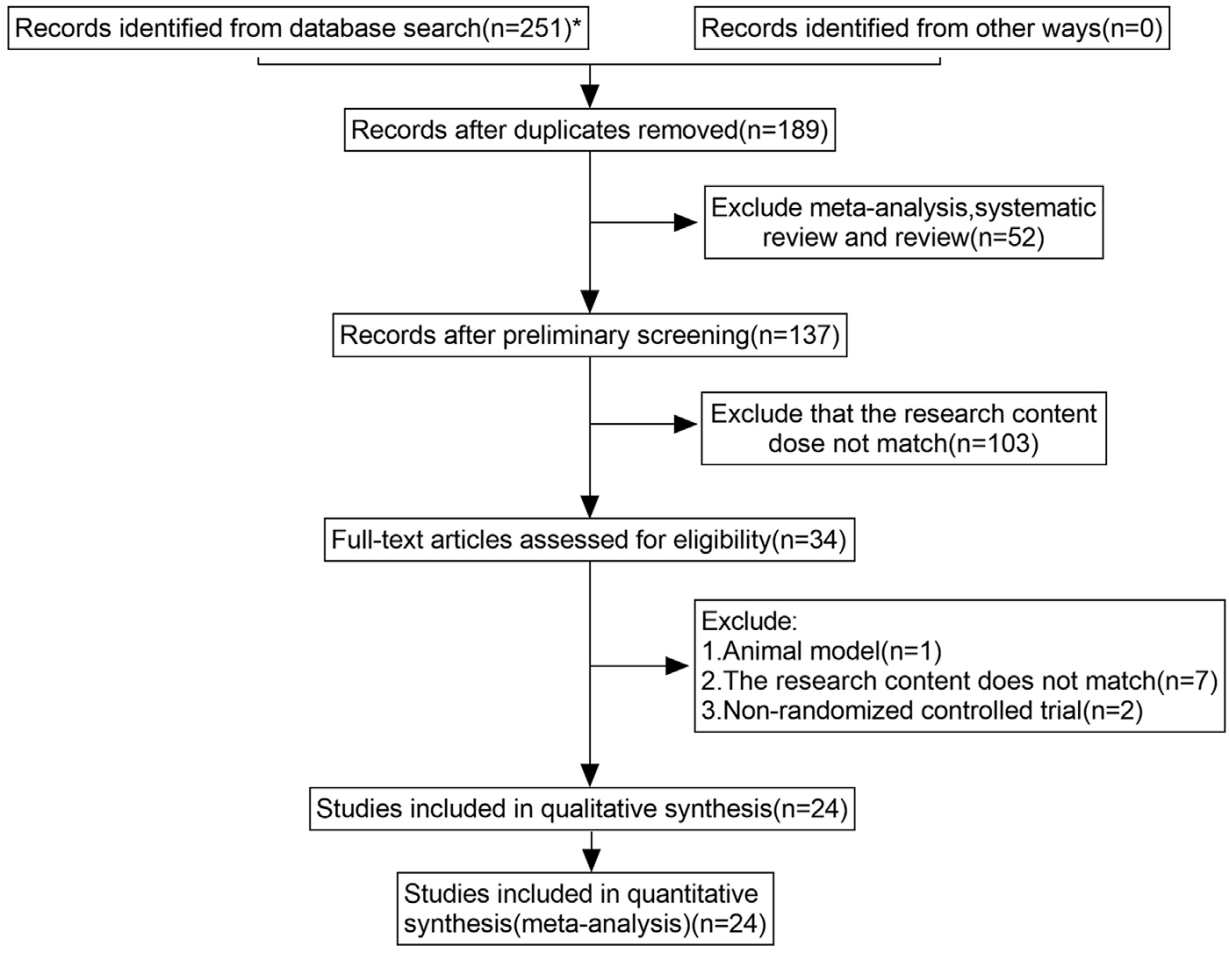
Study selection process for the meta-analysis.

JZ conducted a comprehensive database search. Two reviewers (QW, XDM) independently screened titles and abstracts for eligibility. If there was disagreement, consensus was reached through discussion. Studies were excluded at this stage only if the available information in the title or the abstract clearly indicated ineligibility. Full texts of the remaining articles were retrieved and independently reviewed by the two reviewers. A consensus meeting was held to address any disagreement. The process for selecting studies is illustrated in Figure 1.

### Assessment of risk of bias

Two independent reviewers will assess the risk of bias for each included RCT using the Cochrane Collaboration’s risk of bias tool (Higgins et al., 2011). The risk of bias will be critically assessed across seven domains: random sequence generation, allocation concealment, blinding of participants and personnel, blinding of outcome assessment, incomplete outcome data, selective reporting and other sources of bias. This assessment will involve categorizing the risk of bias as “low,” “high,” or “unclear.” We will exclusively include RCTs that employ rigorously described randomisation procedures, ensuring that only those with a low risk of bias in random sequence generation are considered for inclusion. Any disagreements will be resolved through discussion or by reaching a consensus with a third reviewer. The graphical representation of the assessment of risk of bias will be produced using RevMan V.5.4.

### Data extraction

Two reviewers independently performed article selection and data extraction. Discrepant results were discussed until consensus was reached. The initial set of studies was selected based on title and abstract screening, followed by full-text analysis for confirmation. In cases of multiple records for the same trial, only the earliest publication with the most comprehensive reporting was included. Excluded studies after full-text analysis were documented with reasons for exclusion. Data was extracted into an Excel spreadsheet. Data for the calculation of effect size for primary outcomes (PFS-R and QLQ-C30) and secondary outcomes (KPS score, effective rate, TCM syndrome score, and assessment of adverse reactions) from the first time point after the end of the period of the intervention under assessment in the specific study were extracted by one reviewer (QW) and verified by a second reviewer (XDM). Change values were extracted when both the mean and standard deviation (SD) of the changes were available, or when the available data permitted their calculation. When change values were unavailable or uncalculable, the mean and standard deviation (SD) of post-treatment values were extracted. When SDs were unavailable, various approaches were employed to estimate them, including utilizing *p*-values, confidence intervals (CIs), and extracting data directly from figures. When only the median and interquartile ranges (IQRs) are reported, the mean can be approximated by the median, and the standard deviation (SD) can be estimated by dividing the IQR by 1.35 (Cumpston et al., 2022). The effective sizes were transformed by reversing the signs of the means, ensuring that higher values consistently represented increased fatigue (Cheung and Vijayakumar, 2016). If reported numeric data was insufficient for effect size calculation or if non-numeric data was missing, the reviewers contacted the authors.

### Summary of evidence

We will utilize the GRADE methodology to assess the quality of evidence and present concise “Summary of Findings” tables. The “Summary of Findings” tables will be produced utilizing the GRADE working group’s software, specifically GRADEpro or GRADEpro GDT, accessible at www.gradepro.org (Higgins et al., 2011). The content of the “Summary of Findings” tables, which encompass key outcomes pertinent to patients and decision-makers, will be determined by the aforementioned review group. Whenever feasible, both relative and absolute measures of effect will be included. The GRADE approach assesses the quality of evidence, categorizing it as “high,” “moderate,” “low,” or “very low,” based on the outcome. Evidence may be downgraded in its category due to concerns regarding risk of bias, imprecision, inconsistency, indirectness, or publication bias. Conversely, it may be upgraded if it demonstrates a large effect size, plausible confounding that could alter the effect size, or a clear dose-response relationship. Reviewers will adjust the evidence rating based on the GRADE criteria outlined in Chapter 11 of the Cochrane Handbook, considering both the anticipated effects and the differences within the primary group of interest (Higgins et al., 2011; Lin and Chu, 2018). The overall quality of the evidence will be evaluated based on the assessments of both individual reviewers and the collective decision of the review board.

### Statistical analysis

The statistical software Stata 18.0 (Stata Corp., College Station, TX, United States) and Review Manager 5.4 (Nordic Cochran Centre, Copenhagen, Denmark) were utilized for the statistical analyses. Heterogeneity among the studies was assessed using Cochrane’s Q test and I2 statistics (Migliavaca et al., 2022). If *p* > 0.1 or I2<50%, a fixed effects model was employed for the meta-analysis; otherwise, a random effects model was used. The Mantel–Haenszel method will be used to pool dichotomous data, presenting results as risk ratios (RR) with 95% confidence intervals (CIs). The inverse variance method will be used to pool continuous data, presenting results as standardized mean differences (SMD) with 95% Cis (Kuritz et al., 1988). A two-tailed *p*-value of <0.05 was considered statistically significant.

Publication bias was assessed using funnel plots and Begg’s test when 4 or more studies were included in the meta-analysis. If publication bias was present, a trim-and-fill method was used to adjust the estimates from unpublished studies, and the adjusted results were compared with the original pooled RR (Lin, 2020). Sensitivity analysis was conducted to assess the impact of each individual study on the combined results by systematically removing one study at a time from the pooled analysis (Hegedus et al., 2012).

### Collection of effective ingredients and targets of BZYQ

The TCMSP database (https://tcmspw.com/tcmsp.php) was utilized to identify the chemical components of different drugs in BZYQ (Ru et al., 2014). This study employed the criteria of oral bioavailability (OB) ≥ 20% and drug-likeness (DL) ≥ 0.1 as screening conditions for effective ingredients (35) (Ru et al., 2014). The target proteins of the active drugs were compared with those in the TCMID and DrugBank databases, and subsequently standardized to human genes using the UniProt database (Consortium, 2023).

### Collection of CRF targets

The targets for cancer-related fatigue (CRF) were obtained from 2 databases, namely, GeneCards (https://www.genecards.org) and OMIM (https://www.omim.org) (Stelzer et al., 2016; Amberger and Hamosh, 2017).

### Intersection target and construction of “BZYQ-CRF” network

The intersection target of BZYQ and CRF was obtained using R language version 4.3.2 (Muley et al., 2023). Herbs, active ingredients, and intersection targets were integrated into Cytoscape 3.10.2 to construct a “BZYQ-CRF” network (Doncheva et al., 2019).

### Analysis of protein-protein interaction

The intersection targets of BZYQ and CRF were input into the STRING database (https://string-db.org/) (Szklarczyk et al., 2023). Protein interaction data with a confidence level (score >0.4) was selected and saved in a TSV format file. The information of node1, node2, and combination scores from the file was imported into Cytoscape software to construct a PPI network (Doncheva et al., 2019).

### GO and KEGG enrichment analysis

The “ClusterProfiler” package was used to perform GO and KEGG enrichment analysis on overlapping targets (Yu et al., 2012). The visualization of the enrichment analysis results was carried out using the “ggplot2” package, and the R language version 4.3.2 was used to create the barplot and dotplot (Gustavsson et al., 2022).

## Results

### Search results

The systematic database search and manual search yielded 251 articles. We screened 34 full texts and ultimately included 24 for further qualitative and quantitative analyses (Figure 1). A final list of the 24 articles finally included in the quantitative analysis is provided in Table 2 of Supplementary Figure S4. Subsequently, we conducted an analysis of six outcome indicators, comprising two primary measures (QLQ-C30 and PFS-R) and four secondary measures (KPS score, TCM syndrome score, effective rate, and ADR).

### Patient characteristics

This study included 24 research studies, encompassing a total of 1886 patients, 40 of whom were from South Korea. Among the patients, 989 were male, accounting for approximately 52.44%, and 897 were female, accounting for about 47.56%. The average age of patients experiencing cancer-related fatigue was 56.84 years (ranging from 41 to 80 years). The study involved X types of cancer, including XX and XX, with the remaining types being unspecified. The intervention period of the BZYQ prescription varied from 2 to 12 weeks, comprising 5 studies of 2 or 3 weeks, 7 studies of 4 weeks, and 2 studies of 6, 8, and 12 weeks. Additionally, the study encompassed both BZYQ decoction and BZYQ granules. The characteristics of the included studies are detailed in Table 1.

**TABLE 1:** Clinical information from the eligible trials in the meta-analysis.

CT/CT + BZYQ decoction (OA)	CT/CT + BZYQ decoction (OA)	CT/CT + BZYQ decoction (OA)	CT/CT + BZYQ decoction (OA)	CT/CT + BZYQ decoction (OA)
Jong Soo Jeong 2010	53.4 ± 8.0 49.4 ± 10.8	CT/CT + BZYQ decoction extract granule (OA)	2 weeks	⑥
高可新2021	62.24 ± 6.85 61.73 ± 6.49	CT/CT + BZYQ decoction (OA)	1 month	① ④⑤⑥
梅莎莎2023	65.86 ± 7.37 66.15 ± 6.94	CT/CT + BZYQ granule (OA)	1 month	—
苏羚子2023	56.0 + 7.18 66.15 + 7.97	CT/CT + BZYQ granule (OA)	3 weeks	⑥
陈志成2017	57.64 ± 5.18 58.03 ± 5.22	CT/CT + BZYQ decoction (OA)	3 weeks	⑥
谢燕华2020	48.56 ± 3.41 48.52 ± 3.44	CT/CT + BZYQ decoction (OA)	2 weeks/course, 2 course	② ⑥
于建华2019	58.78 ± 5.98 58.07 ± 6.36	CT/CT + BZYQ decoction (OA)	3 weeks	③⑥
杨昌卫2018	75.01 ± 10.61 74.89 ± 10.18	CT/CT + BZYQ decoction (OA)	1 month	②③
谭刚2021	55.89 ± 4.41 56.17 ± 4.29	CT/CT + BZYQ decoction combined with Shashen Maidong decoction (OA)	12 weeks	②
王欣2023	56.23 ± 2.56 56.43 ± 2.37	CT/CT + BZYQ decoction and Siwu decoction (OA)	2 weeks	①④
段春燕2021	40∼78 41∼78	CT/CT + BZYQ decoction (OA)	1 month	①②
梁冰2024	60.13 ± 8.08 58.29 ± 7.83	CT/CT + BZYQ decoction combined with Ling turtle eight methods (OA)	8 weeks	① ④⑤
张新庆2022	53.62 ± 9.45 52.98 ± 9.86	CT/CT + BZYQ decoction and Gua Sha syndrome differentiation (OA)	1 month	①②
李欣欣2023	48.02 ± 7.38 47.85 ± 7.41	CT/CT + BZYQ decoction (OA)	2 weeks	③ ⑤⑥
陈诗园2020	49.83 ± 13.02 48.20 ± 12.44	CT/CT + BZYQ decoction (OA)	1 month	③
李恩强2022	51.71 ± 8.31 51.08 ± 8.27	CT/CT + BZYQ decoction (OA)	3 months	①⑤
林振荣2018	54∼76 55∼74	CT/CT + BZYQ decoction (OA)	2 weeks	⑤
姜爱萍2022	63.16 ± 7.29 61.62 ± 3.39	CT/CT + BZYQ decoction (OA)	1 month	②
朱国栋2016	—	CT/CT + BZYQ decoction (OA)	56 days	①
王国华2022	55.30 ± 2.05 59.30 ± 2.50	CT/CT + BZYQ decoction (OA)	1 month	③
刘海洋2022	40.66 ± 3.66 41.11 ± 3.69	CT/CT + BZYQ decoction (OA)	42 days	②⑥
陈 宁2017	54.3 ± 5.7 54.6 ± 5.5	CT/CT + BZYQ decoction (OA)	3 weeks	—
宁博彪2020	61.2 ± 9.0 61.0 ± 12.5	CT/CT + BZYQ decoction (OA)	12 weeks	① ②④⑤
季尹霞2021	48.50 ± 6.84 50.39 ± 7.02	CT/CT + Meridian flow theory under the guidance of mild moxibustion and BZYQ decoction (OA)	2 weeks	① ④⑤

Notes: Control group: conventional treatments alone group; Experimental group: Conventional treatments and BZYQ, prescription combined group.

① KPS, score; ② Piper fatigue modified scales; ③ QLQ-C30, quality of Life score; ④ TCM, syndrome score; ⑤ Effective rate; ⑥ Adverse reactions.

Abbreviations: CT, conventional treatments; OA, oral administration.

### Intervention and control conditions

The baseline sample comprised 1886 participants, with 939 assigned to the control group and 947 to the intervention group. The mean duration of interventions was 5.8 weeks (range, 2–12). Regarding control interventions, 22 studies (91.7%) used standard cancer care, no intervention, or waitlist control, while 2 studies (8.3%) used auricular pressure as a control. All included studies reported nationally and internationally accepted diagnostic criteria for malignancy and cancer-related fatigue. Additionally, 9 studies reported KPS scores, 8 reported Piper fatigue modified scales, 6 reported TCM syndrome scores, 4 reported QLQ-C30 quality of life scales, 7 reported TCM effective rates, and 8 reported adverse reactions. The mean and SD for all outcome measures, including both primary and secondary outcomes, are presented in Table 4 of Supplementary Figure S6. Additionally, the effective rate and adverse reaction outcomes are detailed in Table 5 of Supplementary Figure S7.

### Quality assessment

**FIGURE 2:**
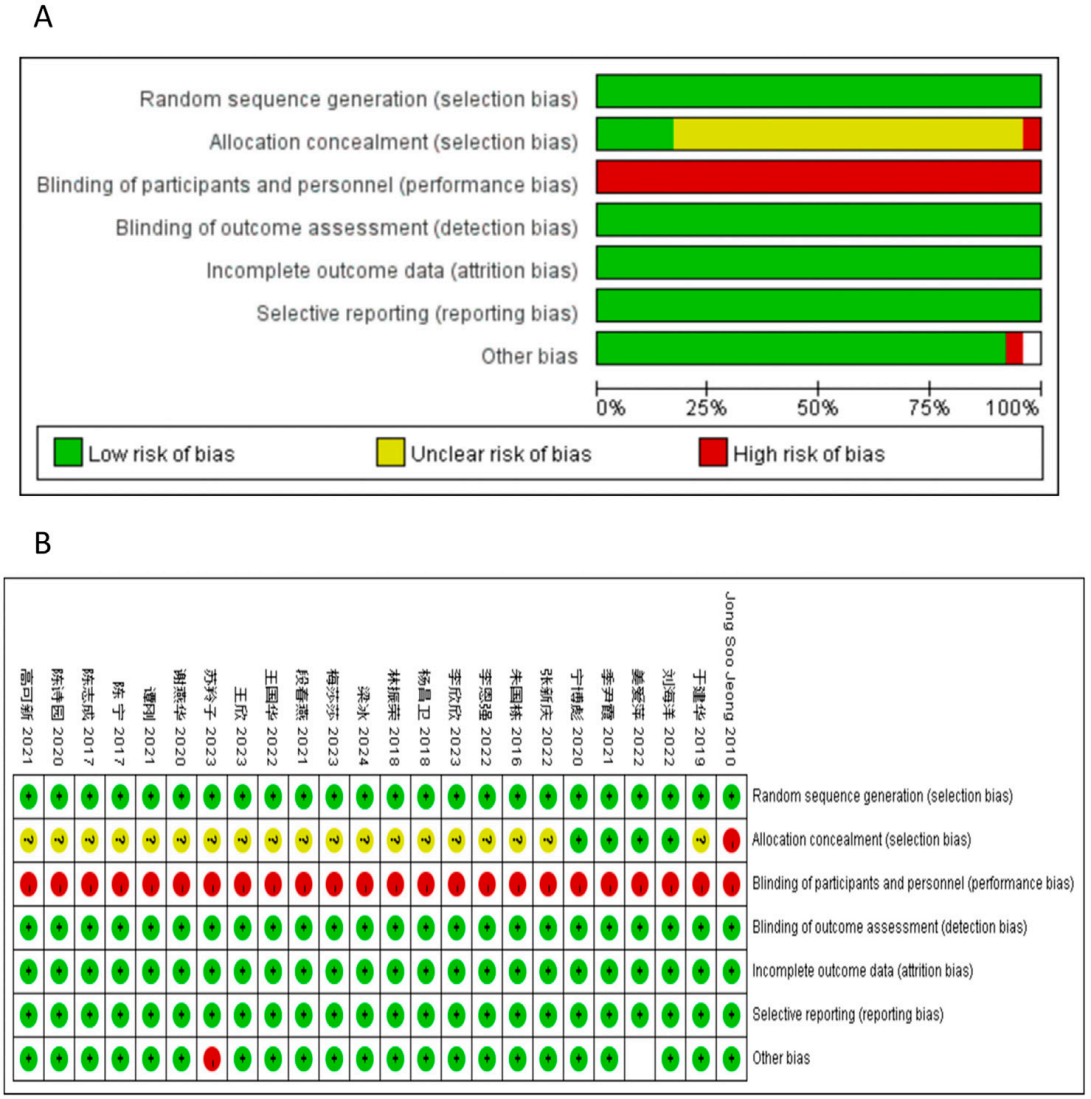
Risk of bias. Review of authors’ judgements about each risk of bias item for included studies. **(A)** bar plot of risk of bias. The composition ratio of different risks in each field of all included studies is shown. **(B)** Summary of risk of bias. The risk of bias of each included study in each field and the grade evaluation results of the overall risk of bias are shown. Note: Each color represents a different level of bias: read for high-risk, green for low-risk, and yellow for unclear-risk of bias.

All the studies included in this analysis were randomized and controlled. 9 utilized the random number table method, 2 utilized the lottery method, and the remaining studies did not specify the randomization process. Quality assessment of the risk of bias is shown in Figure 2; Table 3 in Supplementary Figure S5. The results indicated that the article retrieved for this study exhibited a medium to high level of quality, as presented in Table 6 of Supplementary Figure S8.

### Therapeutic efficacy assessments

#### KPS score

**FIGURE 3:**
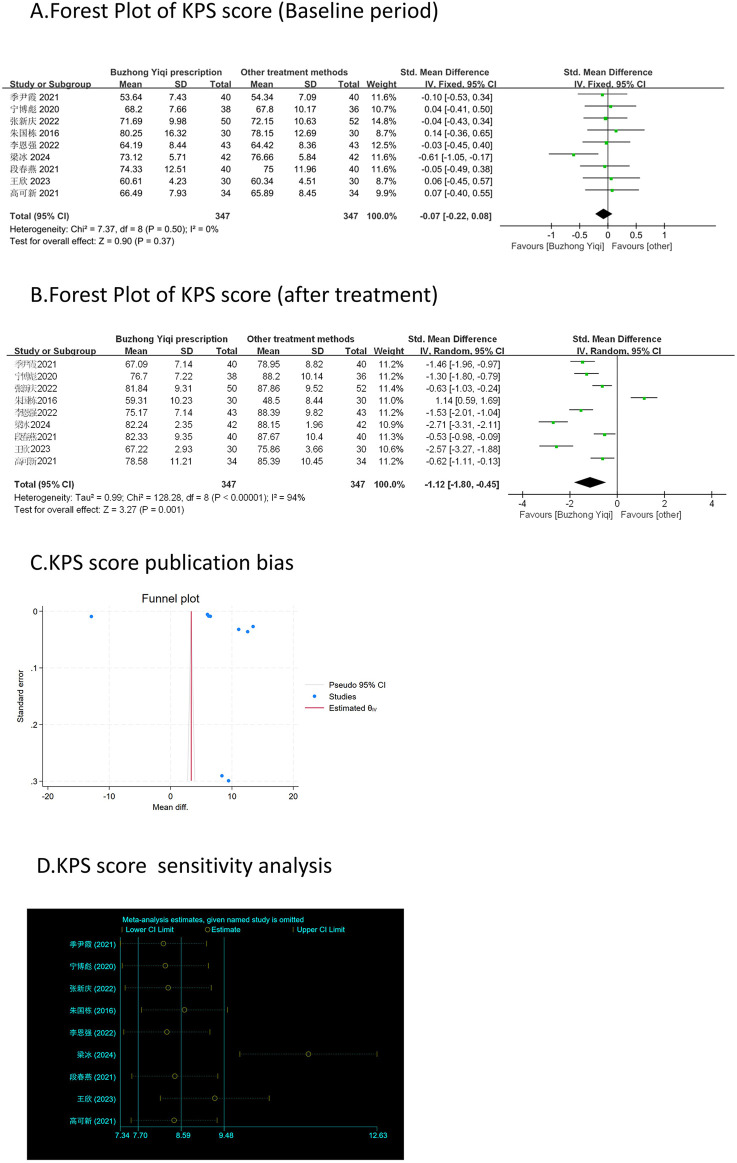
Comparisons of KPS score between experimental and control group. **(A)** The forest plot shows the comparison of KPS scores between the experimental group and the control group in the baseline period; **(B)** The forest plot shows a comparison of KPS scores between the experimental and control groups after BZYQ treatment; **(C)** Funnel plot of KPS score publication bias; **(D)** Sensitivity analysis for KPS score.

Nine clinical trials, involving 694 patients, were conducted to compare the KPS scores between the two groups. The pooled results, as depicted in Figures 3A, B, indicated that patients who underwent BZYQ prescription therapy showed an improvement in KPS score (RR = 1.12, 95%CI = 0.45–1.80, *p* = 0.001) compared to those who received conventional treatments alone. The KPS score (*p* < 0.00001, I2 = 94%) exhibited heterogeneity among the studies, thus a random-effect model was employed for the analysis of RR, while a fixed-effect model was used otherwise.

#### Piper fatigue modified scales

**FIGURE 4:**
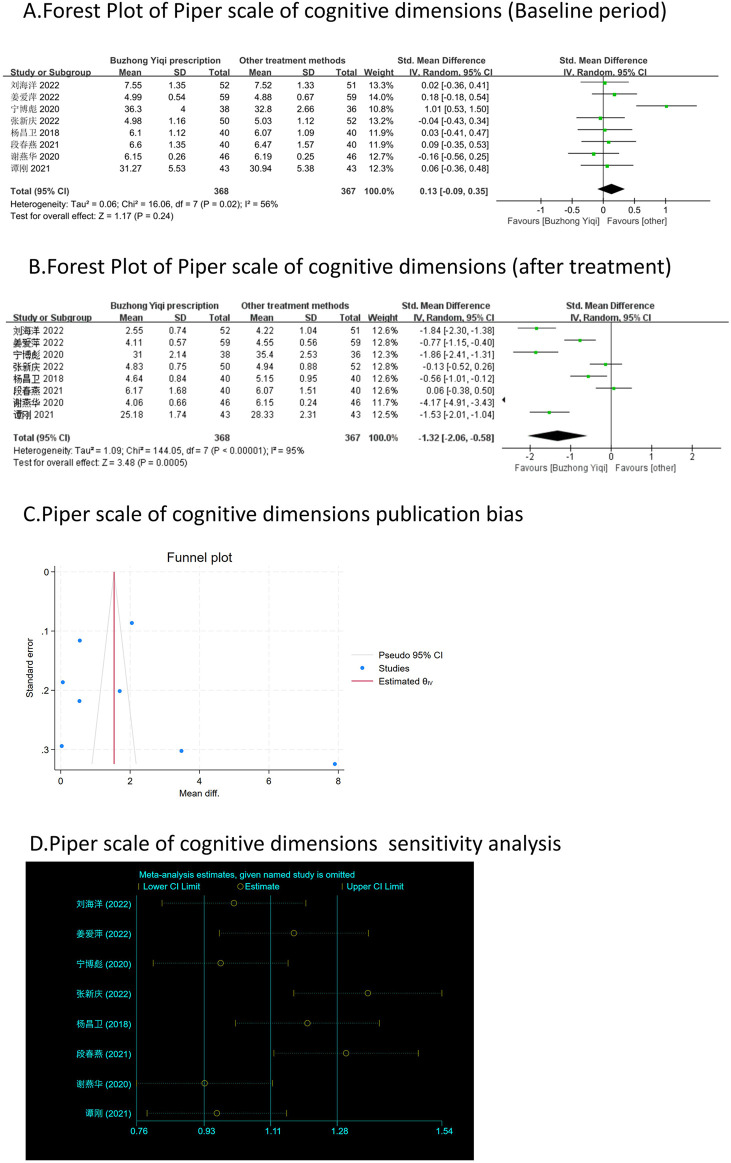
Comparisons of Piper scale of cognitive dimensions between experimental and control group. **(A)** The forest plot shows the comparison of Piper scale of cognitive dimensions between the experimental group and the control group in the baseline period; **(B)** The forest plot shows a comparison of Piper scale of cognitive dimensions between the experimental and control groups after BZYQ treatment; **(C)** Funnel plot of Piper scale of cognitive dimensions publication bias; **(D)** Sensitivity analysis for Piper scale of cognitive dimensions.

**FIGURE 5:**
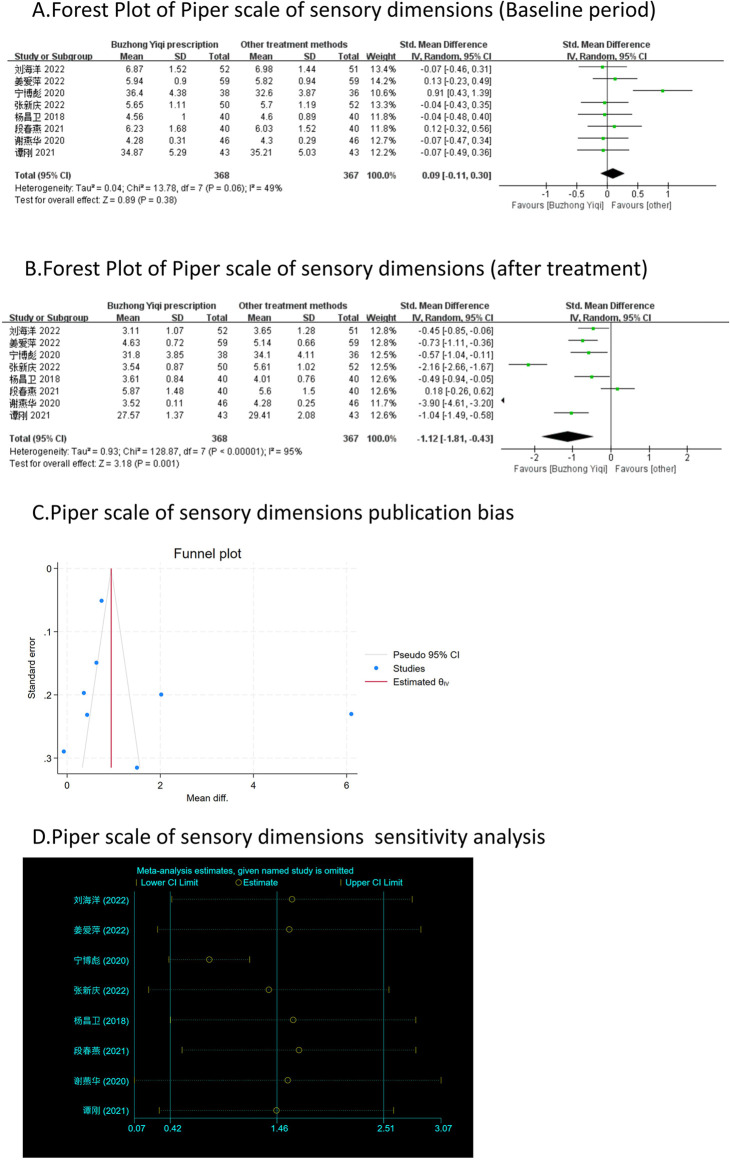
Comparisons of Piper scale of sensory dimensions between experimental and control group. **(A)** The forest plot shows the comparison of Piper scale of sensory dimensions between the experimental group and the control group in the baseline period; **(B)** The forest plot shows a comparison of Piper scale of sensory dimensions between the experimental and control groups after BZYQ treatment; **(C)** Funnel plot of Piper scale of sensory dimensions publication bias; **(D)** Sensitivity analysis for Piper scale of sensory dimensions.

**FIGURE 6:**
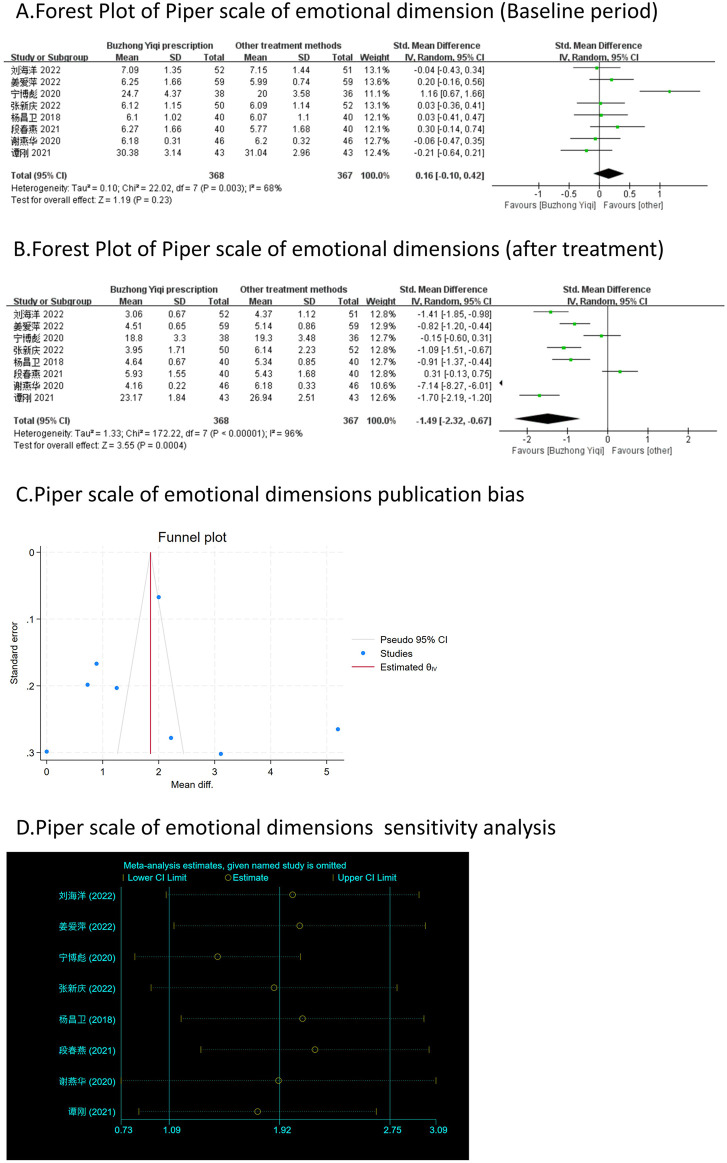
Comparisons of Piper scale of emotional dimension between experimental and control group. **(A)** The forest plot shows the comparison of Piper scale of emotional dimension between the experimental group and the control group in the baseline period; **(B)** The forest plot shows a comparison of Piper scale of emotional dimension between the experimental and control groups after BZYQ treatment; **(C)** Funnel plot of Piper scale of emotional dimensions publication bias; **(D)** Sensitivity analysis for Piper scale of emotional dimensions.

**FIGURE 7:**
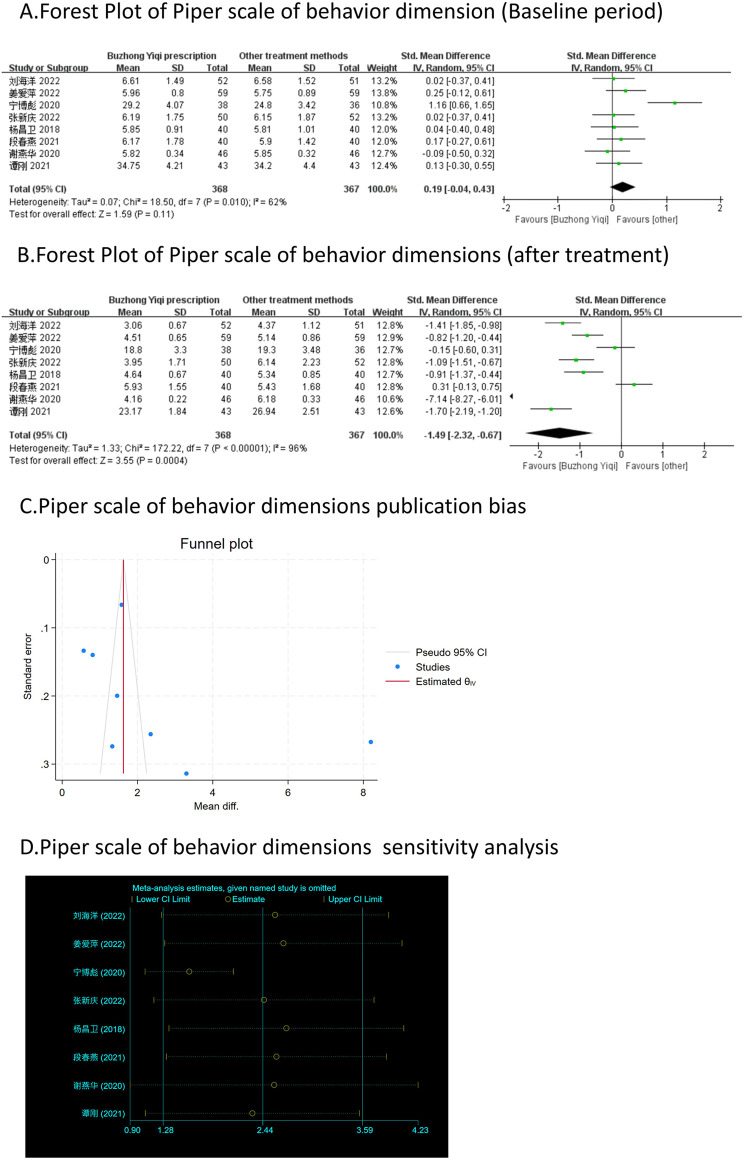
Comparisons of Piper scale of behavior dimension between experimental and control group. **(A)** The forest plot shows the comparison of Piper scale of behavior dimension between the experimental group and the control group in the baseline period; **(B)** The forest plot shows a comparison of Piper scale of behavior dimension between the experimental and control groups after BZYQ treatment; **(C)** Funnel plot of Piper scale of behavior dimensions publication bias; **(D)** Sensitivity analysis for Piper scale of behavior dimensions.

Eight clinical trials, involving 735 patients, compared the modified Piper Fatigue Scale between two groups. As depicted in Figures 4–7A, B, the scores for cognitive, sensory, emotional, and behavioral aspects of the Piper Fatigue Scale decreased significantly (*p* < 0.05) after treatment with the BZYQ prescription, compared to the baseline results of the patients.

### QLQ-C30 quality of life score

**FIGURE 8:**
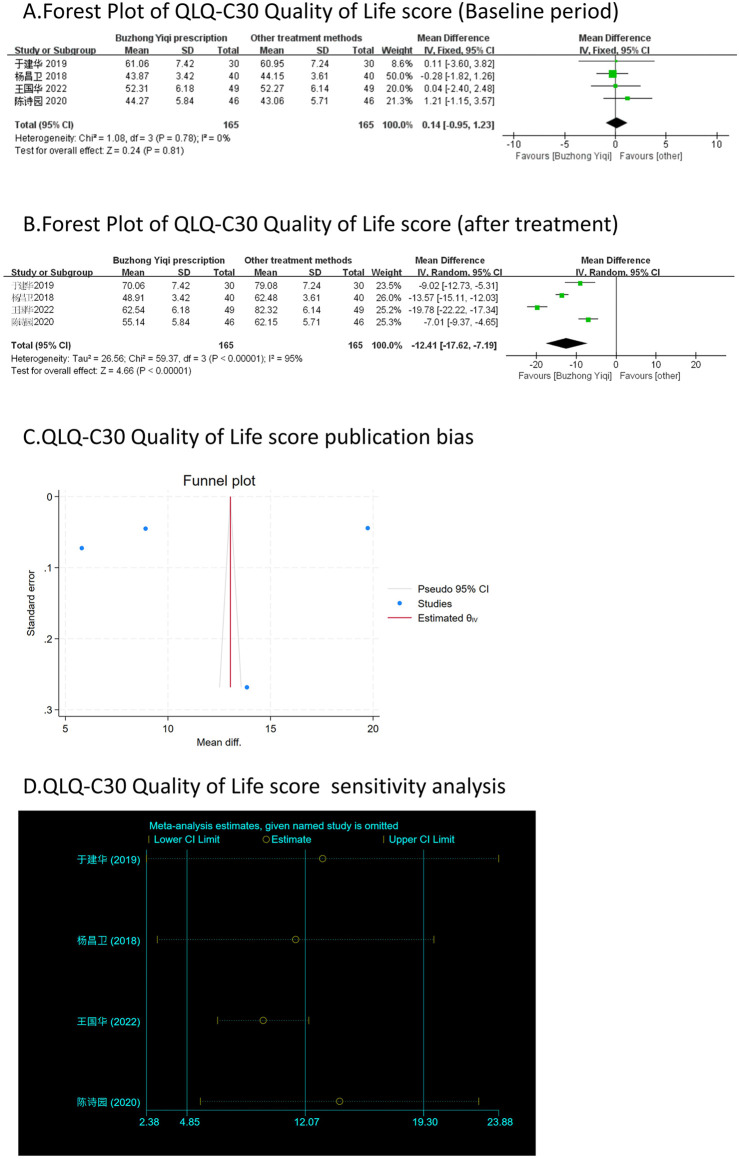
Comparisons of QLQ-C30 Quality of Life score between experimental and control group. **(A)** The forest plot shows the comparison of QLQ-C30 quality of life score between the experimental group and the control group in the baseline period; **(B)** The forest plot shows a comparison of QLQ-C30 Quality of Life score between the experimental and control groups after BZYQ treatment; **(C)** Funnel plot of QLQ-C30 Quality of Life score publication bias; **(D)** Sensitivity analysis for QLQ-C30 Quality of Life score.

Four clinical trials, involving 330 patients, compared the QLQ-C30 quality of life scores between two groups. As depicted in Figures 8A, B, there was no significant difference in the QLQ-C30 scores of the patients at baseline (*p* > 0.05). However, the QLQ-C30 scores of the patients significantly increased after treatment with the BZYQ prescription (*p* < 0.05), indicating an improvement in the patients’ quality of life to a certain extent.

### Effective rate and TCM syndrome score

**FIGURE 9:**
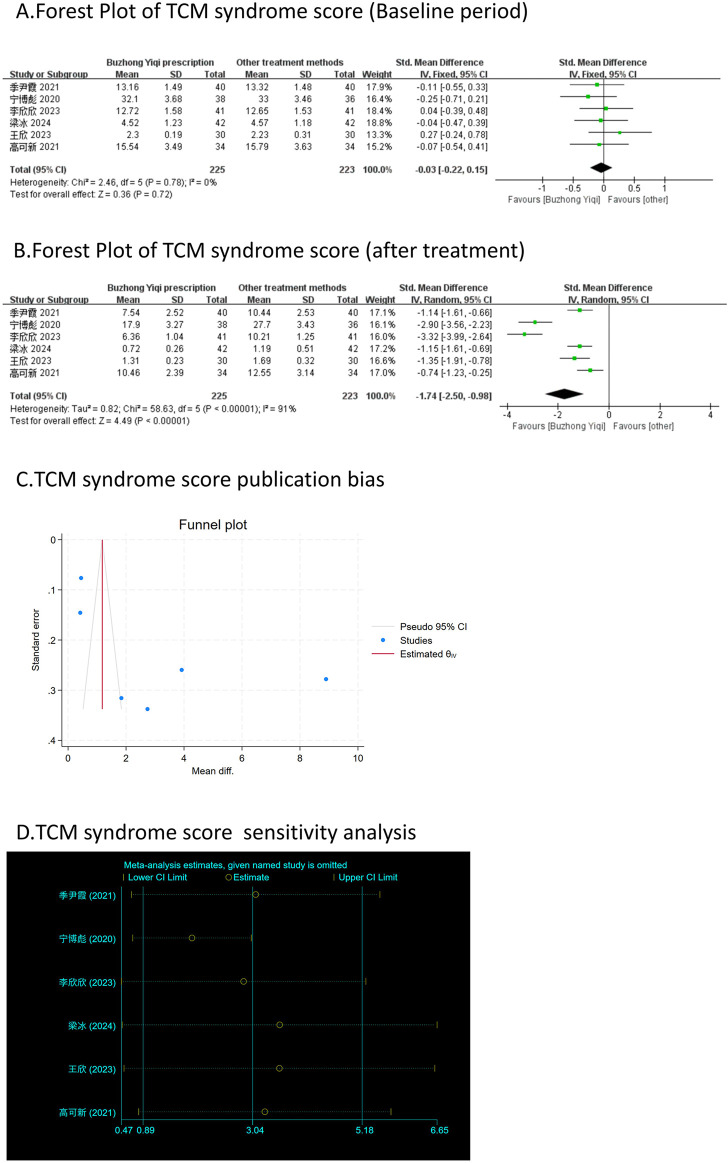
Comparisons of TCM syndrome score between experimental and control group. **(A)** The forest plot shows the comparison of TCM syndrome score between the experimental group and the control group in the baseline period; **(B)** The forest plot shows a comparison of TCM syndrome score between the experimental and control groups after BZYQ treatment; **(C)** Funnel plot of TCM syndrome score publication bias; **(D)** Sensitivity analysis for TCM syndrome score.

**FIGURE 10:**
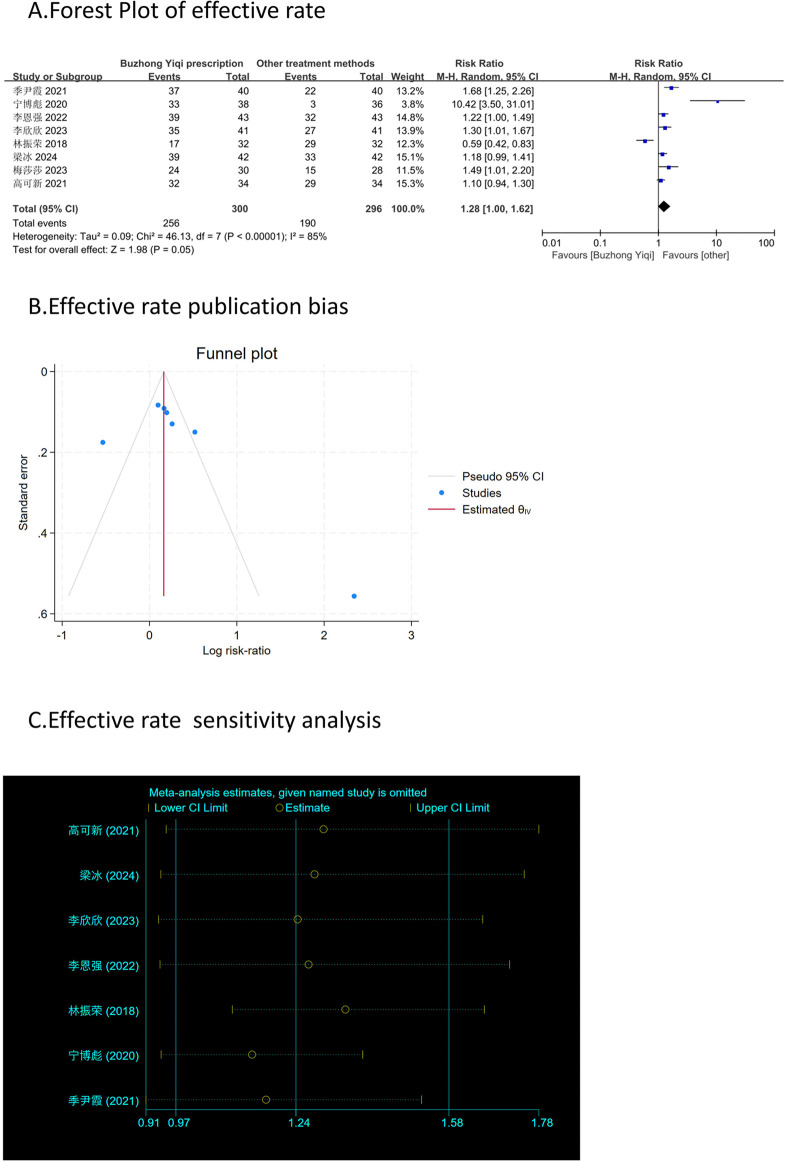
Comparisons of effective rate between experimental and control group. **(A)** The forest plot shows a comparison of effective rate between the experimental and control groups after BZYQ treatment; **(B)** Funnel plot of effective rate publication bias; **(C)** Sensitivity analysis for effective rate.

According to the TCM Syndrome Efficacy Score Scale, the TCM syndrome score is the sum of each pattern. Seven articles, involving 538 patients, only reported the clinical effectiveness rate (Figure 10A), while six articles, involving 448 patients, reported the TCM syndrome scores (Figures 9A, B). After treatment with the BZYQ prescription, the clinical effectiveness rate and TCM syndrome scores of the patients showed a significant increase compared to the baseline results (*p* < 0.05).

### Assessment of adverse reactions

**FIGURE 11:**
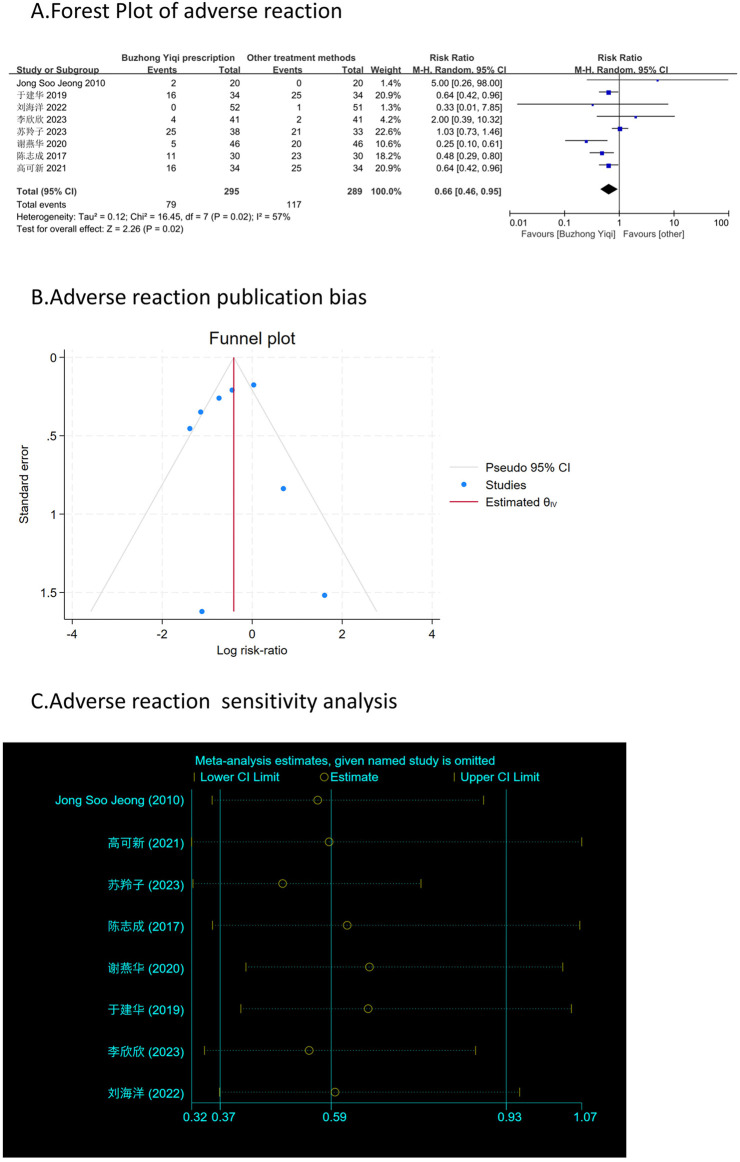
Comparisons of adverse reaction between experimental and control group. **(A)** The forest plot shows a comparison of adverse reaction between the experimental and control groups after BZYQ treatment; **(B)** Funnel plot of adverse reaction publication bias; **(C)** Sensitivity analysis for adverse reaction.

Figure 11A shows that patients treated with the BZYQ prescription and conventional methods had lower incidences of adverse reactions (RR = 0.66, 95% CI = 0.46–0.95), indicating a statistically significant difference between the two groups (*p* < 0.05). The heterogeneity test revealed statistical heterogeneity in adverse reactions (*p* = 0.02, I2 = 57%), leading to the use of a random effects model for pooling this meta-analysis, while a fixed-effect model was used otherwise.

### Publication bias

Figures 3–9C, 10, 11B shows that the funnel plots and Begg’s regression tests results indicated no publication bias in the effective rate (Begg = 0.1331), adverse reactions (Begg = 0.9015), KPS score (Begg = 0.0763), cognitive aspect of the Piper Fatigue Scale (Begg = 0.2655), sensory aspect of the Piper Fatigue Scale (Begg = 0.5362), emotional aspect of the Piper Fatigue Scale (Begg = 0.7105), behavioral aspect of the Piper Fatigue Scale (Begg = 0.1078), TCM syndrome score (Begg = 0.2597), and QLQ-C30 quality of life score (Begg = 0.8241).

### Sensitivity analysis

As shown in Figures 3–9D, 10, 11C, sensitivity analyses were performed for all studies, and the results showed that one study was heterogeneous in terms of KPS scores. In terms of Piper Fatigue Scale, heterogeneity was not significant after one study was excluded. In terms of QLQ-C30 quality of life score, one study was excluded, and the heterogeneity was not significant. No individual studies significantly affected the rest indicators, which indicated statistically robust results.

### Active ingredients screening

The TCMSP database identified the active components of the drug through ADME screening. It revealed 20 huangqi, 22 renshen, 17 chaihu, 2 danggui, 17 shengma, 5 chenpi, 7 baizhu and 101Licorice ingredients. Additionally, the database included 790 target genes for these ingredients.

### CRF targets acquisition

In brief, we obtained 7,570 targets from GeneCards and 318 targets from the OMIM databases.

### “BZYQ-CRF” network construction

**FIGURE 12:**
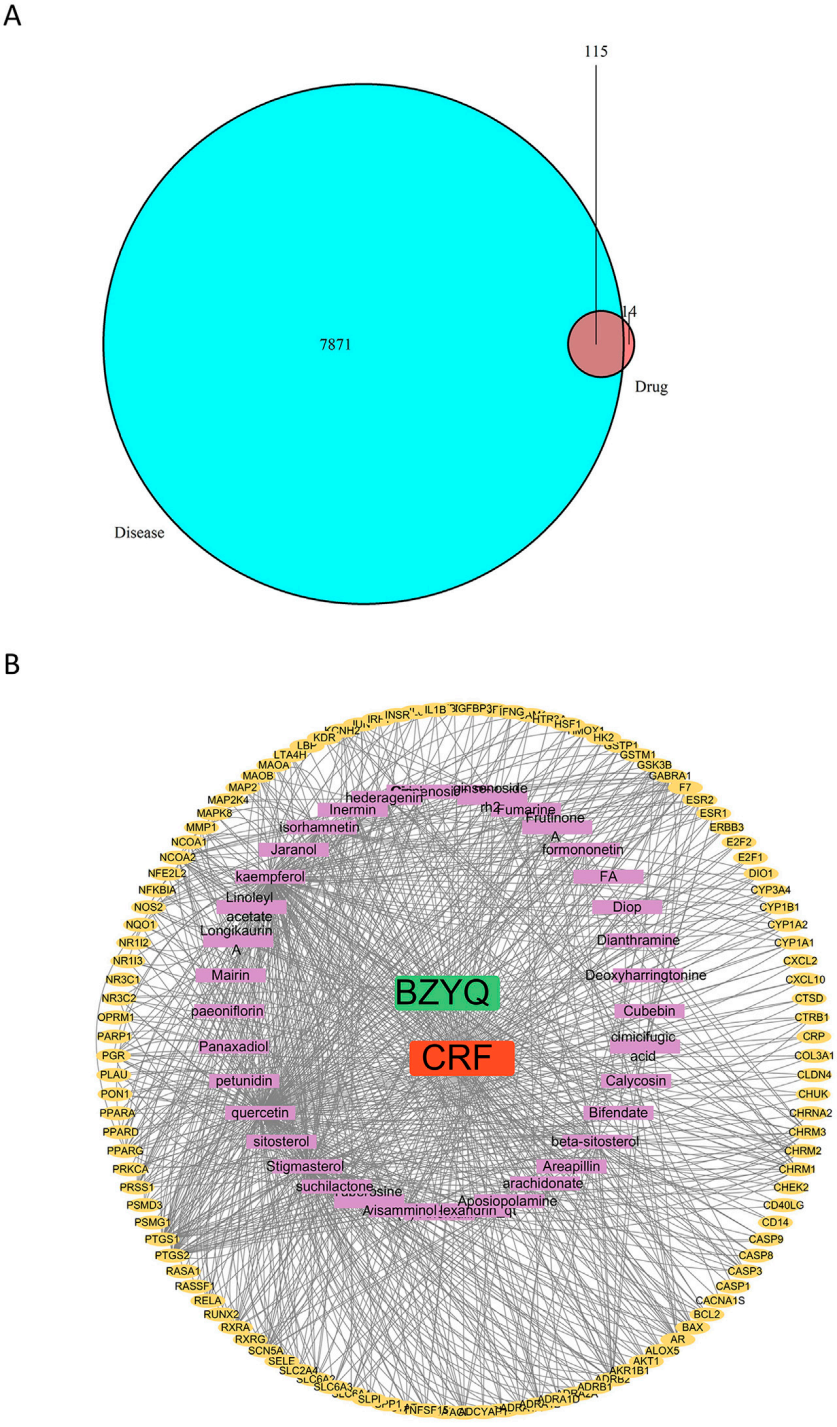
Drug component-target network. **(A)** Venn diagram of the targets of the active ingredients of BZYQ and the CRF-related targets; **(B)** Network diagram of BZYQ components and CRF-related targets plotted with Cytoscape software.

The action targets of BZYQ components were compared with the targets correlated to CRF, resulting in the identification of 115 intersecting targets (Figure 12A). Subsequently, these 115 intersecting targets were imported into Cytoscape 3.8.0 to construct a “BZYQ-CRF” network (Figure 12B). Nodes with higher degree values may be crucial in a network.

### Protein-protein interaction analysis of intersection targets

**FIGURE 13:**
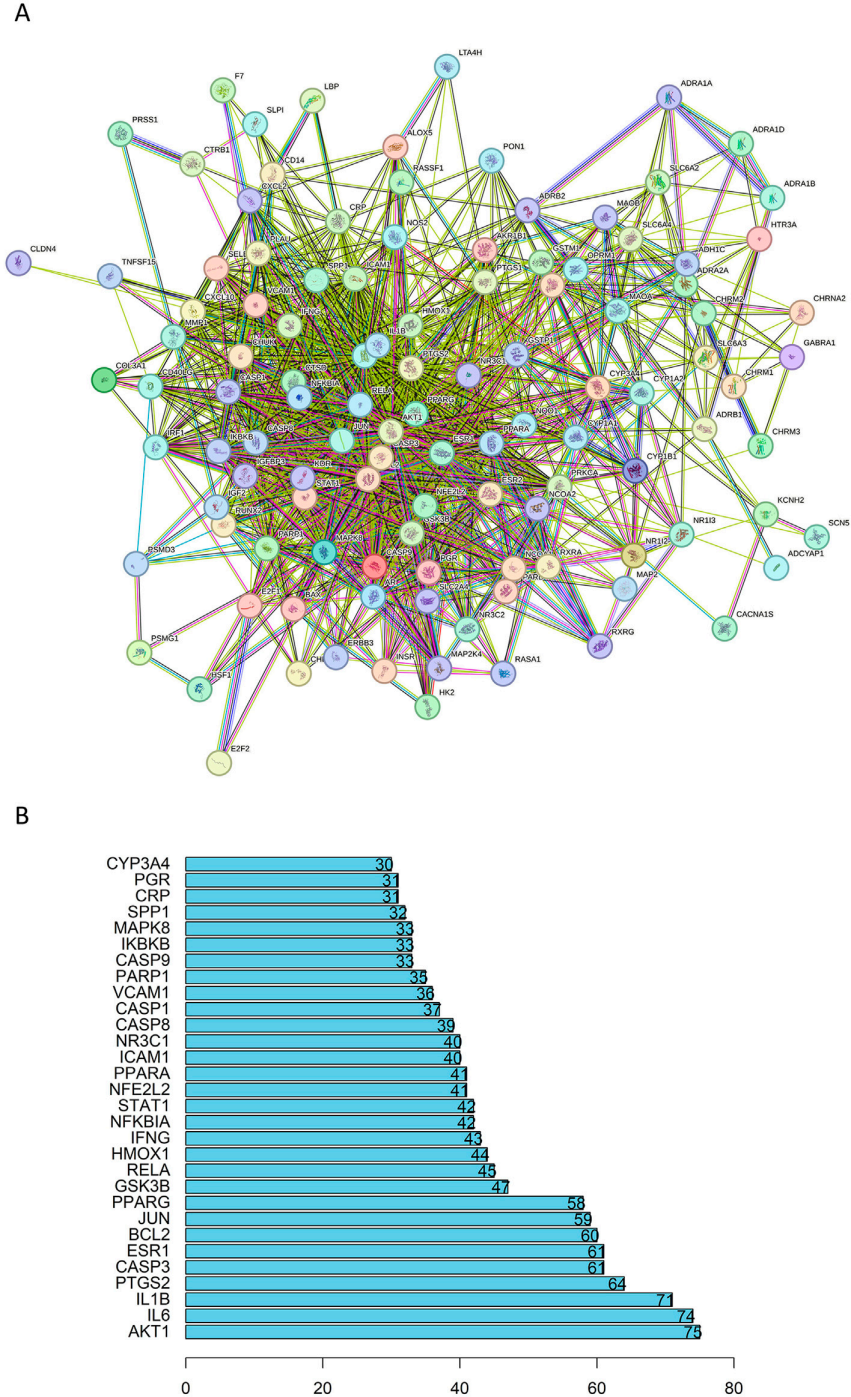
PPI network of the common targets of BZYQ and CRF. **(A)** PPI network diagram plotted on a string network; **(B)** 20 key targets determined in the PPI network.

A total of 115 intersection targets of BZYQ and CRF were uploaded to the STRING 11.0 database (https://www.stringdb.org/) for analysis, resulting in the construction of a PPI network representing the shared targets between BZYQ and CRF. The network consists of 115 nodes, where nodes represent the intersecting targets and edges denote the associations between them. The visualization of the PPI network diagram (Figure 13A) was created using Cytoscape 3.10.2. The top 10 hub genes in the indegree ranking, including AKT1, IL6, IL1B, PTGS2, CASP3, ESR1, BCL2, JUN, PPARG, and GSK3B, were identified (Figure 13B).

### GO and KEGG enrichment analysis of intersecting targets

**FIGURE 14:**
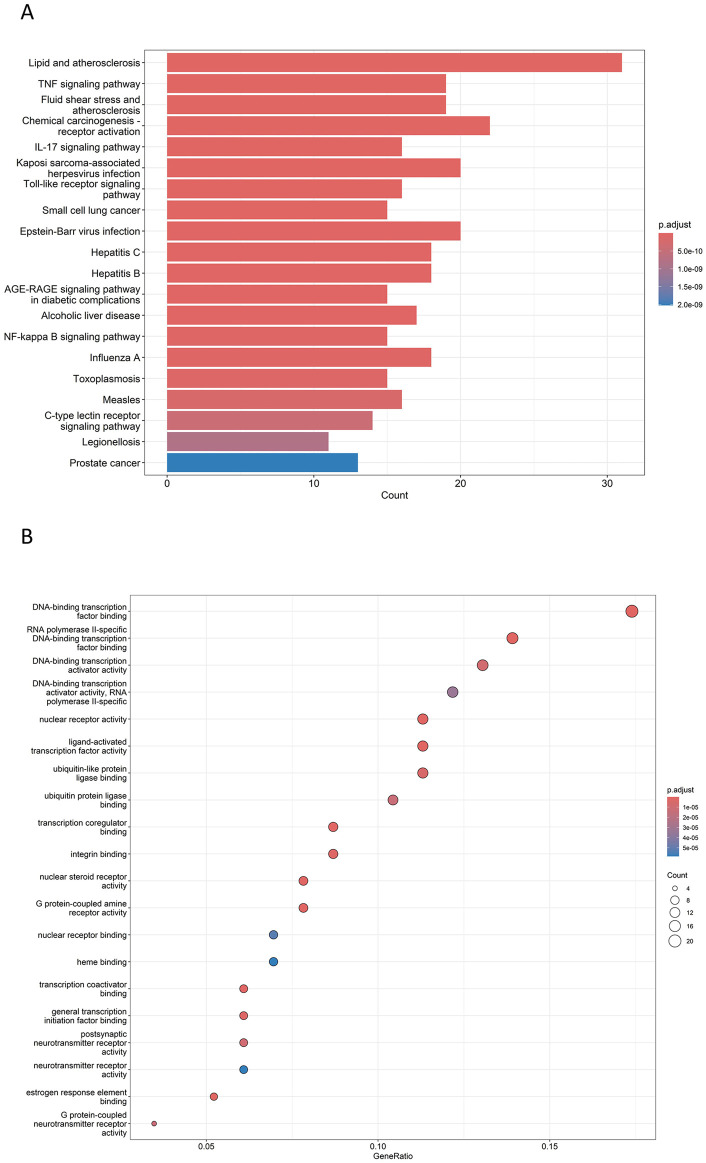
Top GO and KEGG enriched terms of BZYQ in treating CRF. **(A)** Top GO enriched terms of BZYQ in treating CRF. The X-coordinate indicates the number of enriched genes, and the color of the dot represents the *p*-value of the corresponding term. **(B)** Top KEGG enriched pathways of HQSJZD in treating CRF. A bigger dot indicates that more genes are enriched. A bigger dot indicates that more genes are enriched in that pathway, and a dot with a darker red color represents a smaller *p*-value.

**FIGURE 15:**
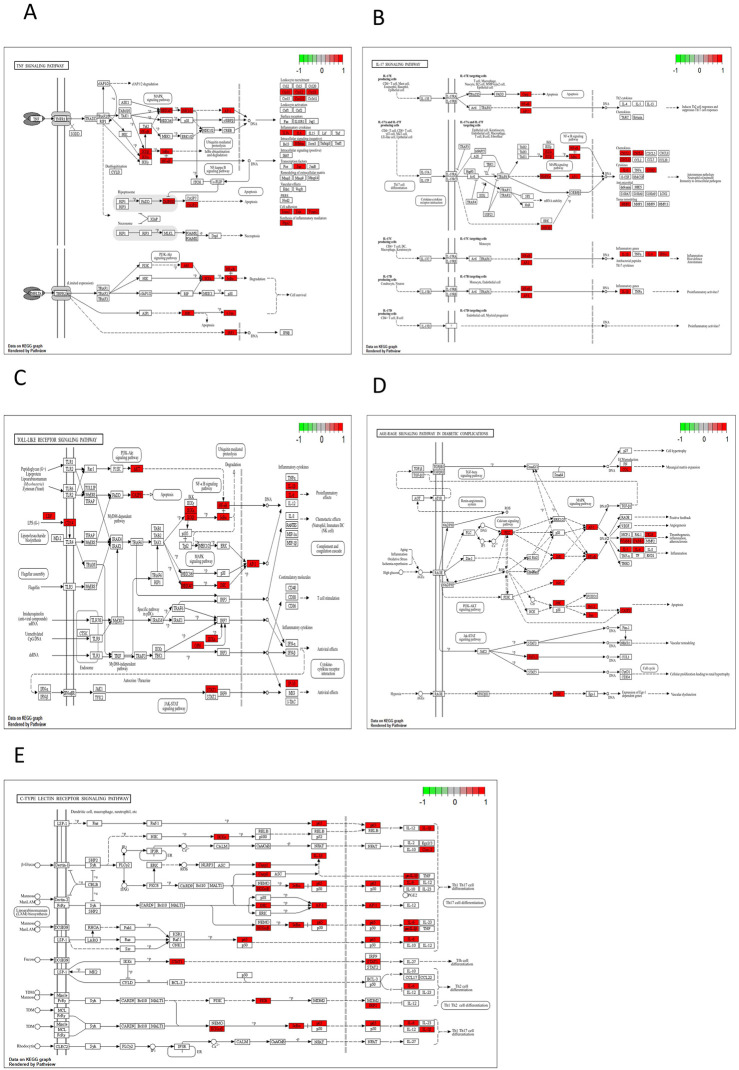
KEGG pathway. **(A)** TNF signaling pathway (*p* = 9.73E-17); **(B)** IL-17 signaling pathway (*p* = 1.10E-12); **(C)** Toll-like receptor signaling pathway (*p* = 7.55E-12); **(D)** AGE-RAGE signaling pathway in diabetic complications (*p* = 2.18E-11); **(E)** C-type lectin receptor signaling pathway (*p* = 3.97E-10).

The key biological functions of BZYQ in treating CRF were determined through GO and KEGG enrichment analysis. The plots of the top 10 biological functions concentrated on molecular function (MF) in GO analysis are presented in Figure 14A. The MF modifications primarily focused on binding with DNA-binding transcription factor, RNA polymerase II transcription factor, DNA-binding transcription activator activity, ubiquitin-like protein ligase, ubiquitin protein ligase, transcription coregulator, and integrin binding. KEGG pathway enrichment analysis identified 138 signal pathways. The analysis indicates that the TNF, IL-17, Toll-like receptor, AGE-RAGE, and C-type lectin receptor signaling pathways could potentially serve as crucial pathways for treating CRF with BZYQ, as illustrated in Figures 14B, 15A–E.

## Discussion

This meta-analysis revealed some heterogeneity among studies utilizing BZYQ interventions for CRF treatment. However, it confirmed that BZYQ significantly improved patients’ fatigue status and effectively enhanced their quality of life. This instills hope in fatigued patients for recovery, encouraging active cooperation with treatment to achieve optimal outcomes. BZYQ has broad potential for clinical application, yet the dosage and treatment duration may need to be adjusted according to the individual’s specific situation.

Traditional Chinese Medicine (TCM) postulates that tumors gradually deplete qi and blood within the body over an extended period. The surgical procedure resulted in significant depletion of the body’s vital energy, commonly referred to as qi; Chemotherapy drugs possess potent emetic properties, which can adversely affect the spleen, stomach qi, and body fluids; The physical radiation used in radiotherapy is intense and potentially harmful, as it can damage bodily fluids and deplete vital energy (qi). Various treatment modalities can significantly deplete the body’s Yin, fluids, and qi of the spleen and stomach, ultimately contributing to cancer-related fatigue (Berger et al., 2015; Thong et al., 2020; Bower, 2014). Astragalus membranaceus served as the primary constituent in the BZYQ prescription, with other components contributing to a synergistic effect. A study investigating the immunomodulatory effects of Astragalus membranaceus, a key component in BZYQ decoction, on spleen-deficient mice revealed that it was the primary agent responsible for enhancing hemoglobin levels (Huang et al., 2017). Subsequent research has verified that Astragalus membranaceus enhances not only the efficacy of IL-2 and LAK activity but also elevates hemoglobin levels and modulates the CD4+/CD8+ ratio (Liu et al., 2024). Previous research has demonstrated that astragalus membranaceus enhances vascular dementia symptoms in mouse models and boosts learning and memory capabilities, suggesting a beneficial impact on cognitive decline in patients with CRF (Tsao et al., 2021). Astragalus membranaceus, ginseng, Atractylodes rhizoma, and Glycyrrhizae radix were employed to fortify qi and strengthen the spleen, thereby enhancing IL-2, NK, and IFN-γ activity in both healthy and spleen-deficient mice (Huang et al., 2017; Dun et al., 2016). Therefore, the BZYQ prescription for treating CRF not only significantly improves patients’ quality of life but also enhances their immunity, promotes bodily recovery, and delays as well as controls tumor progression and metastasis, indicating its high clinical potential value.

Although BZYQ has been shown to be effective in treating CRF, its pharmacological mechanism is still not well understood. Therefore, we also conducted a detailed discussion on the pharmacological mechanism of BZYQ in the treatment of CRF through network pharmacology. This discussion provided a theoretical basis for further research and clinical practice of BZYQ in treating CRF. The network evaluation of BZYQ-CRF revealed the top 5 active compounds in the treatment of CRF by BZYQ, which included Tuberosine A, cimicifugic acid, paeoniflorin, visammiol, 23-epi-26deoxyactein_qt, and stigmasterol.

The current studies indicate that fukinolic acid and cimicifugic acids found in Cimicifuga rhizomes may have the potential to prevent collagen degradation caused by collagenases or collagenolytic enzymes in pathological conditions, wound healing, or inflammation (Kusano et al., 2001). Paeoniflorin, as a key component of traditional Chinese medicine, is known for its broad anti-inflammatory and immune-regulatory effects. Numerous studies have also validated its antidepressant effects (Zhang and Wei, 2020; Wang et al., 2021). Studies have also demonstrated that stigmasterol can ameliorate neuroinflammation in APP/PS1 mice and suppress the microglial inflammatory response to a-β42 oligomers via the AMPK/NF-κB and AMPK/NLRP3 signaling pathways (Jie et al., 2022).

Based on the attribute parameters of the core targets, BZYQ primarily addresses CRF by targeting AKT1, IL6, IL1B, PTGS2, CASP3, ESR1, and BCL2. One study discovered that alterations in the cellular levels of AKT1 result in changes in the levels of a group of differentially expressed genes, which in turn, indicate the resulting cellular functions of AKT1. Another study demonstrates the critical role of AKT1 in tumor angiogenesis (George et al., 2022; Ha et al., 2022). Longitudinal studies have shown that increased fatigue symptoms, particularly in women with early-stage breast cancer, are linked to high levels of neutrophil/monocyte, IL1RA, and IL6 during radiation therapy (Saligan and Kim, 2012). Single nucleotide polymorphisms (SNPs) in several cytokines, such as IL1B, IL-1RN, and IL-10, exhibited significant correlations with fatigue levels in survivors of lung cancer (Rausch et al., 2010). There is insufficient research on the PTGS2, CASP3, ESR1, and BCL2 targets in cancer-induced fatigue. Future investigations involving target prediction, animal experiments, and clinical testing may reveal their potential as new treatment targets for cancer-induced fatigue.

The KEGG analysis of core targets indicated that BZYQ affects multiple signaling pathways related to tumor and inflammation in the treatment of CRF, including TNF, IL-17, Toll-like receptor (TLR), NF-κB, and C-type lectin receptor (CLR) signaling pathways.

A study showed that TLR4^−/−^ mice implanted with both mEER and LLC tumors exhibited reduced expression of inflammatory cytokines. Only TLR4^−/−^ mice implanted with LLC tumors were protected from developing fatigue-like behavior (Vichaya et al., 2020). One study has also found a link between cancer-related fatigue in head and neck cancer (HNC) patients and increased activity of pro-inflammatory NF-kB family transcription factors, as well as decreased activity of innate antiviral IRF family transcription factors in peripheral blood mononuclear cells (PBMC) (Xiao et al., 2018). While some studies have reported CLR-mediated modulation of T2 immune responses to allergens, it remains unclear whether CLR is involved in the disease progression and prognosis of CRF by modulating the process of inflammatory immunity. Further research is required (Angelina et al., 2023).

In summary, BZYQ’s Tuberosine A, cimicifugic acid, paeoniflorin, visammiol, 23-epi-26deoxyactein_qt, and stigmasterol may target AKT1, IL6, IL1B, PTGS2, CASP3, ESR1, and BCL2, as well as TNF, IL-17, and signaling pathways such as TLR, NF-κB, and C-type lectin receptor, playing an active role in treating CRF. Our study not only validated the therapeutic effect of BZYQ on CRF through meta-analysis but also predicted its potential molecular mechanism in treating CRF through network pharmacology. However, due to the lack of validation from animal experiments in our study, further animal experimental studies are necessary to explore its specific molecular mechanism and provide a certain molecular basis for the clinical treatment of CRF.

## Conclusion

BZYQ demonstrates significant efficacy in treating CRF with minimal adverse reactions. It can serve as a fundamental treatment for CRF in clinical practice, and the medication can be tailored to individual patients for personalized therapy. The potential pharmacological mechanism of BZYQ in treating CRF, as predicted by network pharmacology, offers a molecular foundation for clinical CRF treatment.

## Data Availability

The original contributions presented in the study are included in the article/Supplementary Material, further inquiries can be directed to the corresponding author.
