# Supplementary material for: Systematic review of Buzhong Yiqi method in alleviating cancer-related fatigue: a meta-analysis and exploratory network pharmacology approach
Source: Front Pharmacol. 2024 Nov 5;15:1451773. doi: 10.3389/fphar.2024.1451773 (PMC11573511; doi:10.3389/fphar.2024.1451773)
Supplement: Supplementary file 1 [file Presentation1.PPTX]

## Slide 1
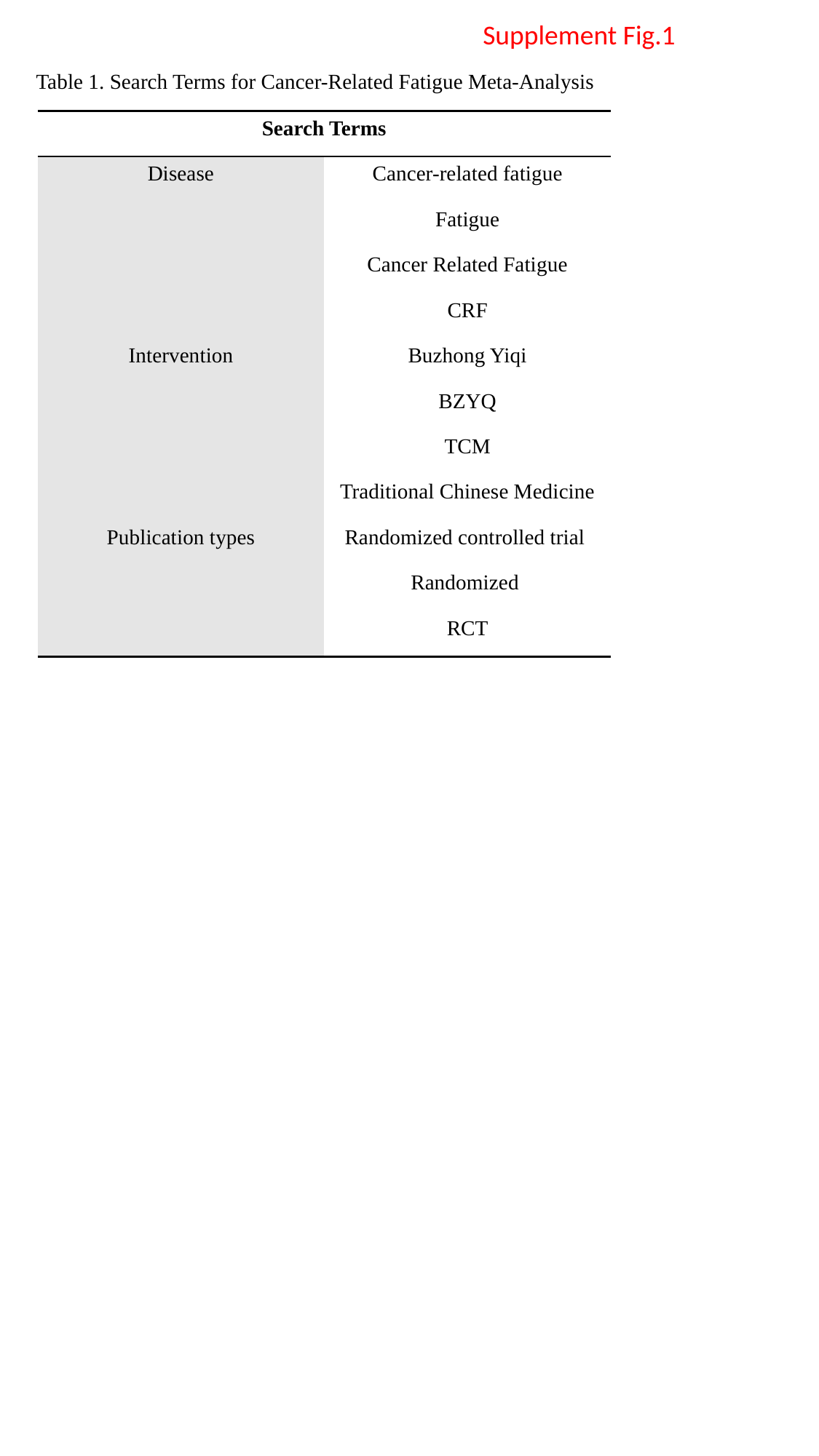

Supplement Fig.1
Table 1. Search Terms for Cancer-Related Fatigue Meta-Analysis
| Search Terms | |
| --- | --- |
| Disease | Cancer-related fatigue |
| | Fatigue |
| | Cancer Related Fatigue |
| | CRF |
| Intervention | Buzhong Yiqi |
| | BZYQ |
| | TCM |
| | Traditional Chinese Medicine |
| Publication types | Randomized controlled trial |
| | Randomized |
| | RCT |

## Slide 2
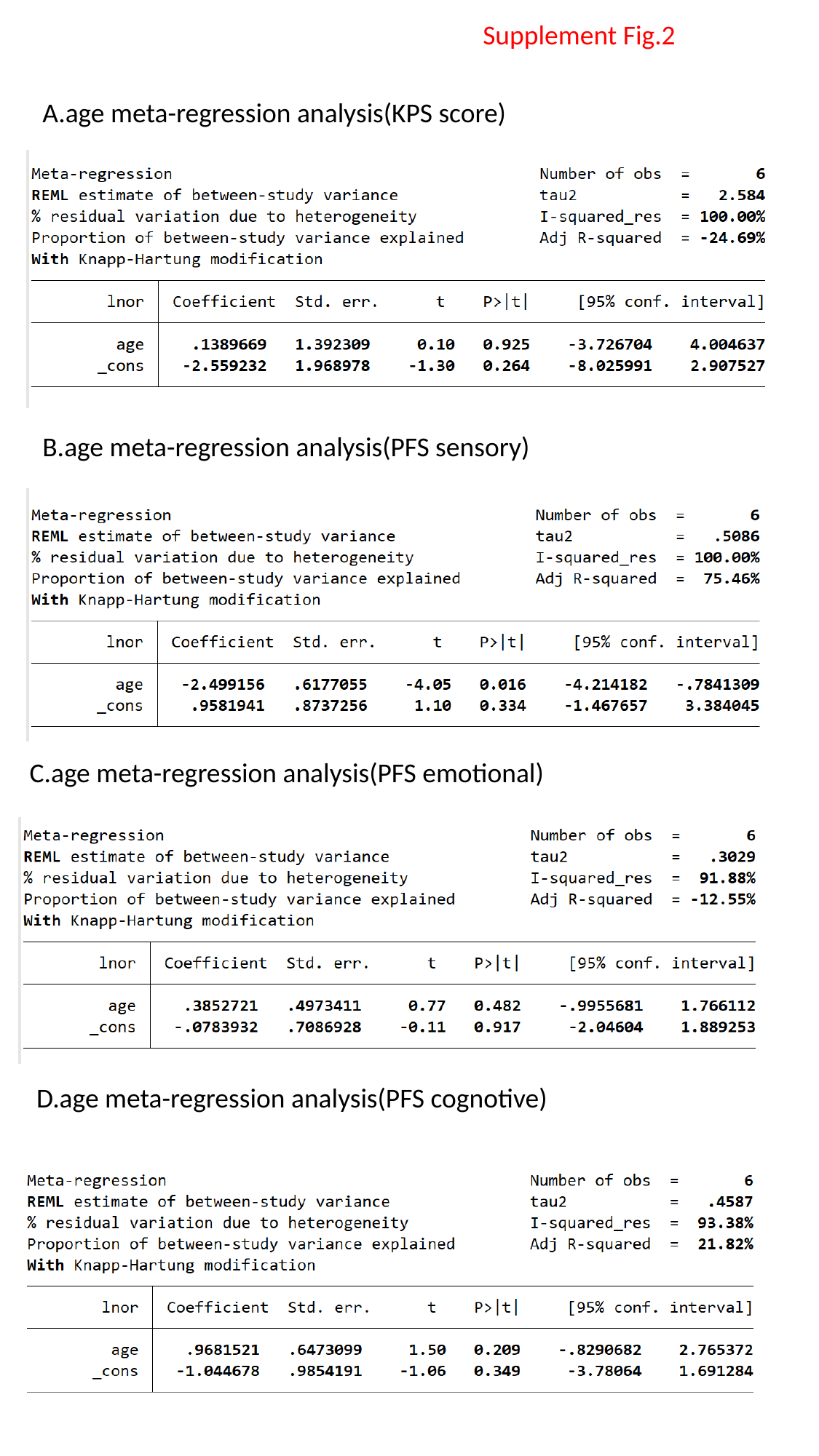

Supplement Fig.2
A.age meta-regression analysis(KPS score)
B.age meta-regression analysis(PFS sensory)
C.age meta-regression analysis(PFS emotional)
D.age meta-regression analysis(PFS cognotive)

## Slide 3
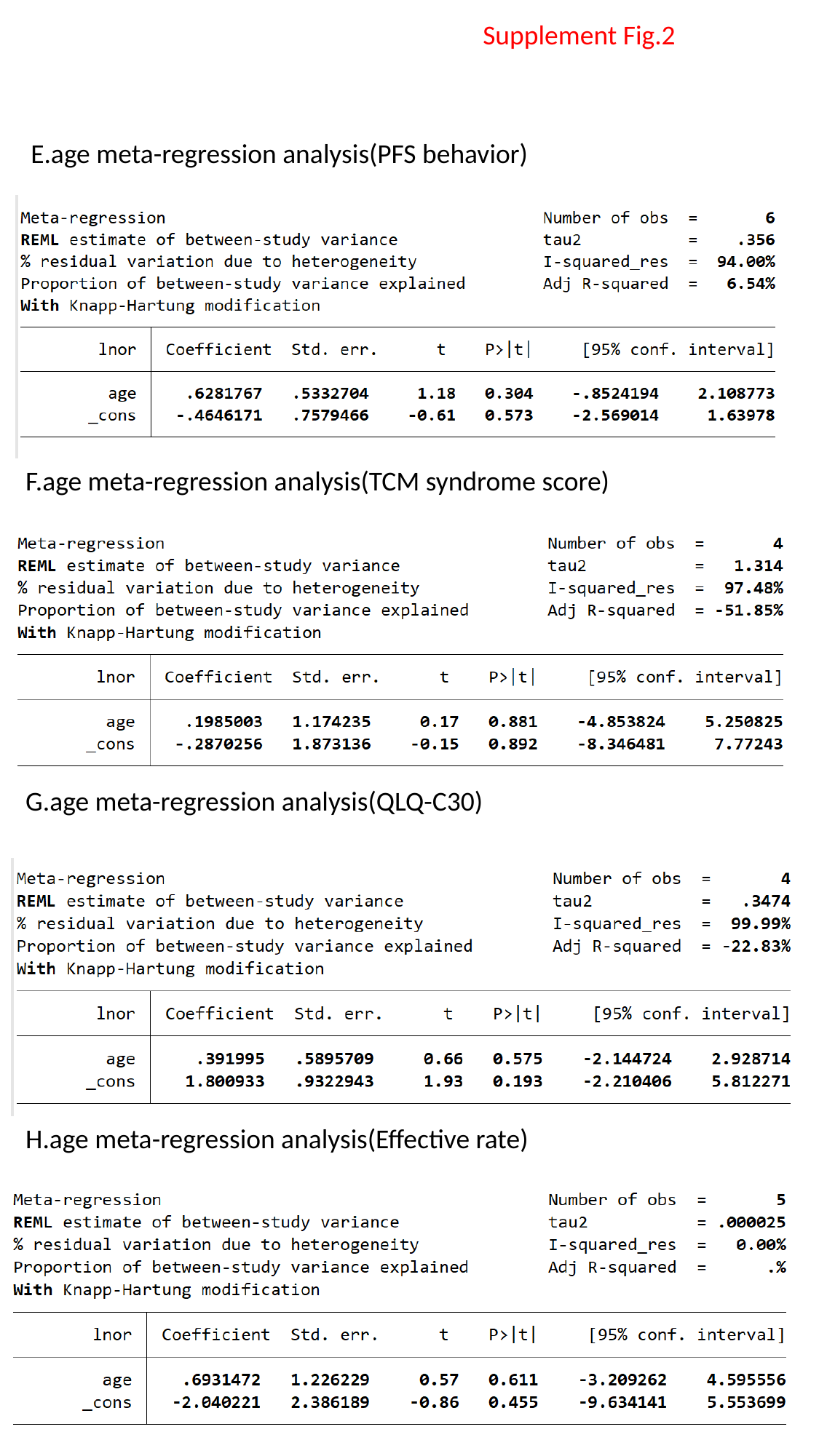

Supplement Fig.2
E.age meta-regression analysis(PFS behavior)
F.age meta-regression analysis(TCM syndrome score)
G.age meta-regression analysis(QLQ-C30)
H.age meta-regression analysis(Effective rate)

## Slide 4
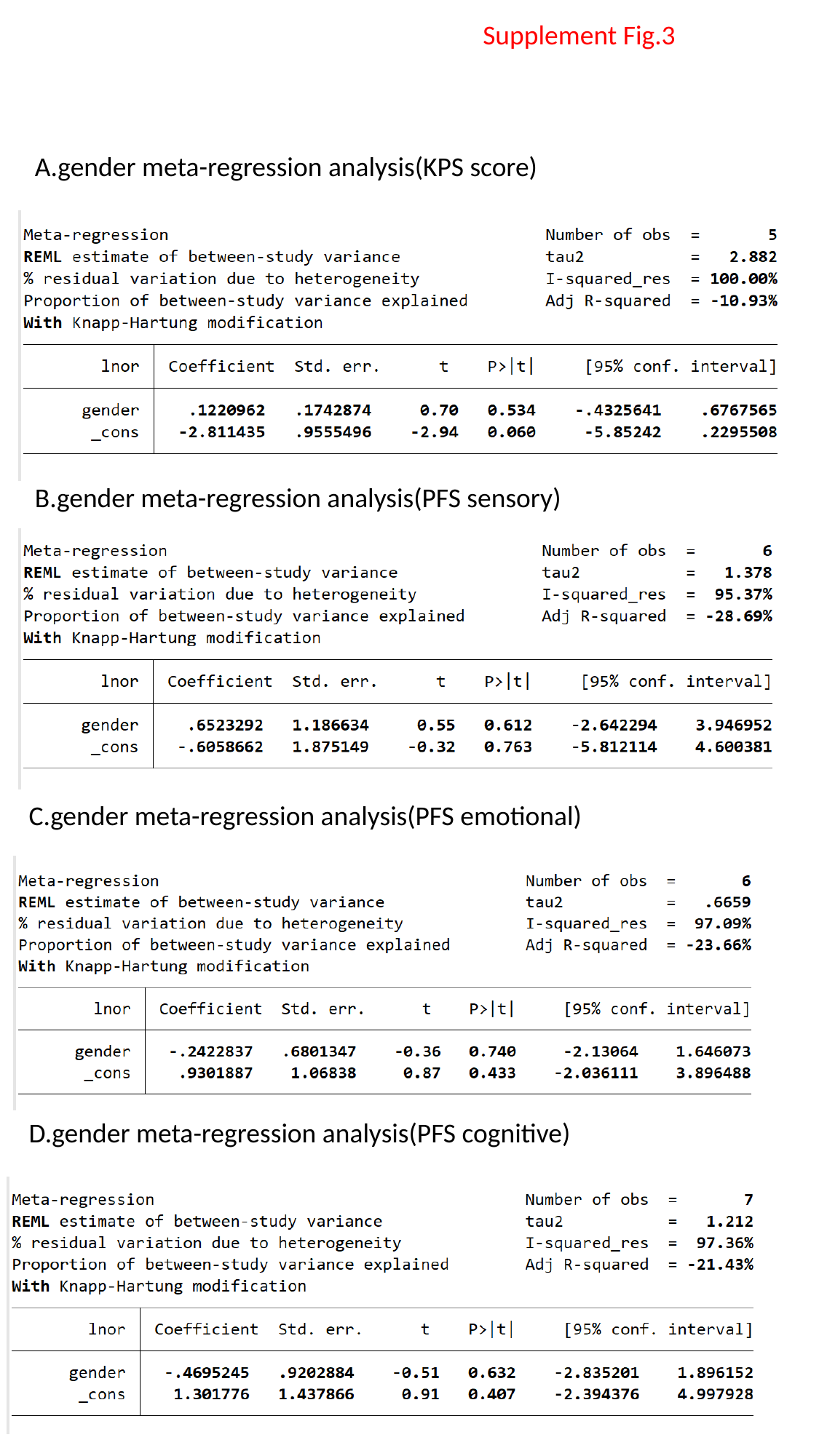

Supplement Fig.3
A.gender meta-regression analysis(KPS score)
B.gender meta-regression analysis(PFS sensory)
C.gender meta-regression analysis(PFS emotional)
D.gender meta-regression analysis(PFS cognitive)

## Slide 5
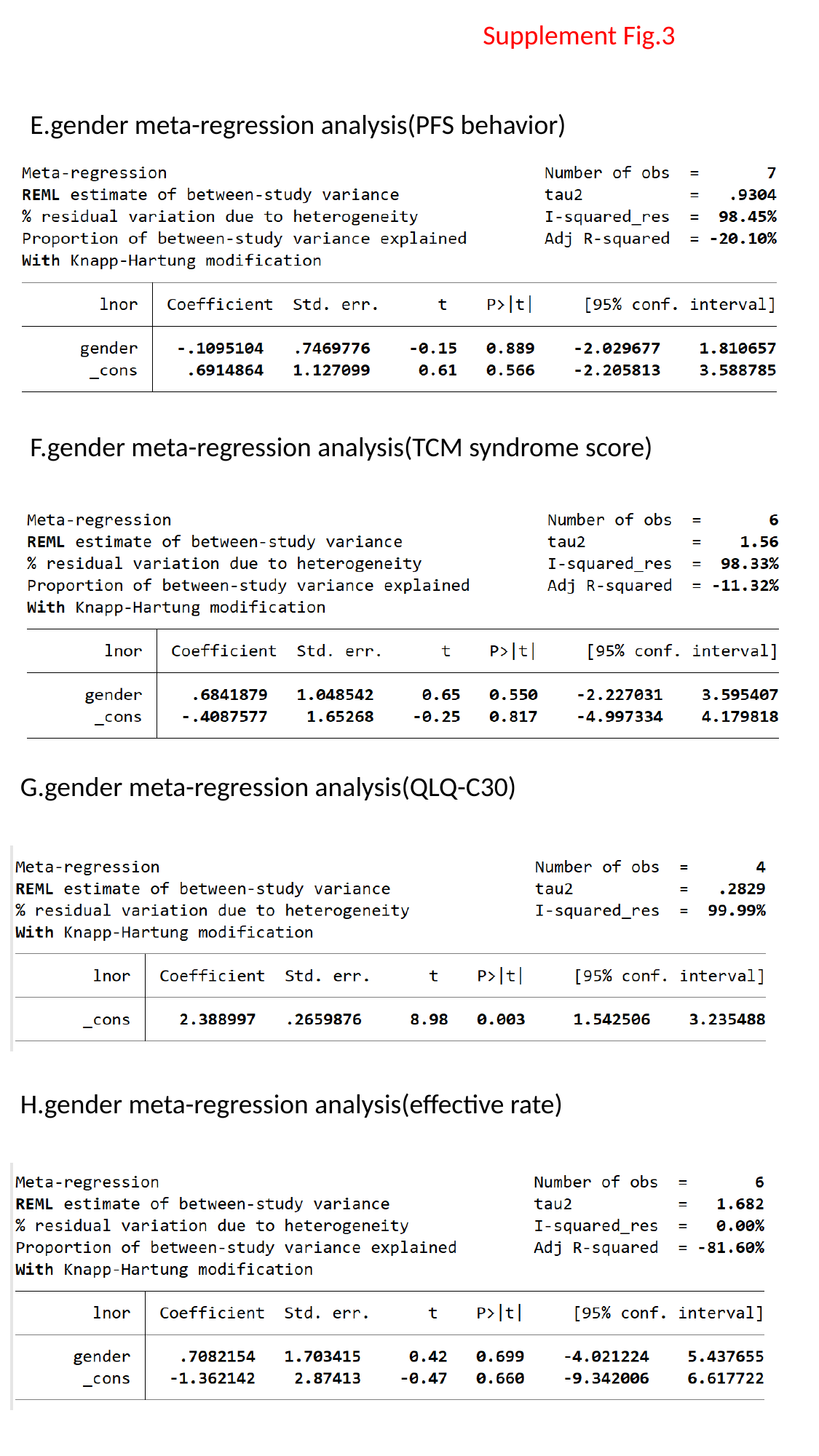

Supplement Fig.3
E.gender meta-regression analysis(PFS behavior)
F.gender meta-regression analysis(TCM syndrome score)
G.gender meta-regression analysis(QLQ-C30)
H.gender meta-regression analysis(effective rate)

## Slide 6
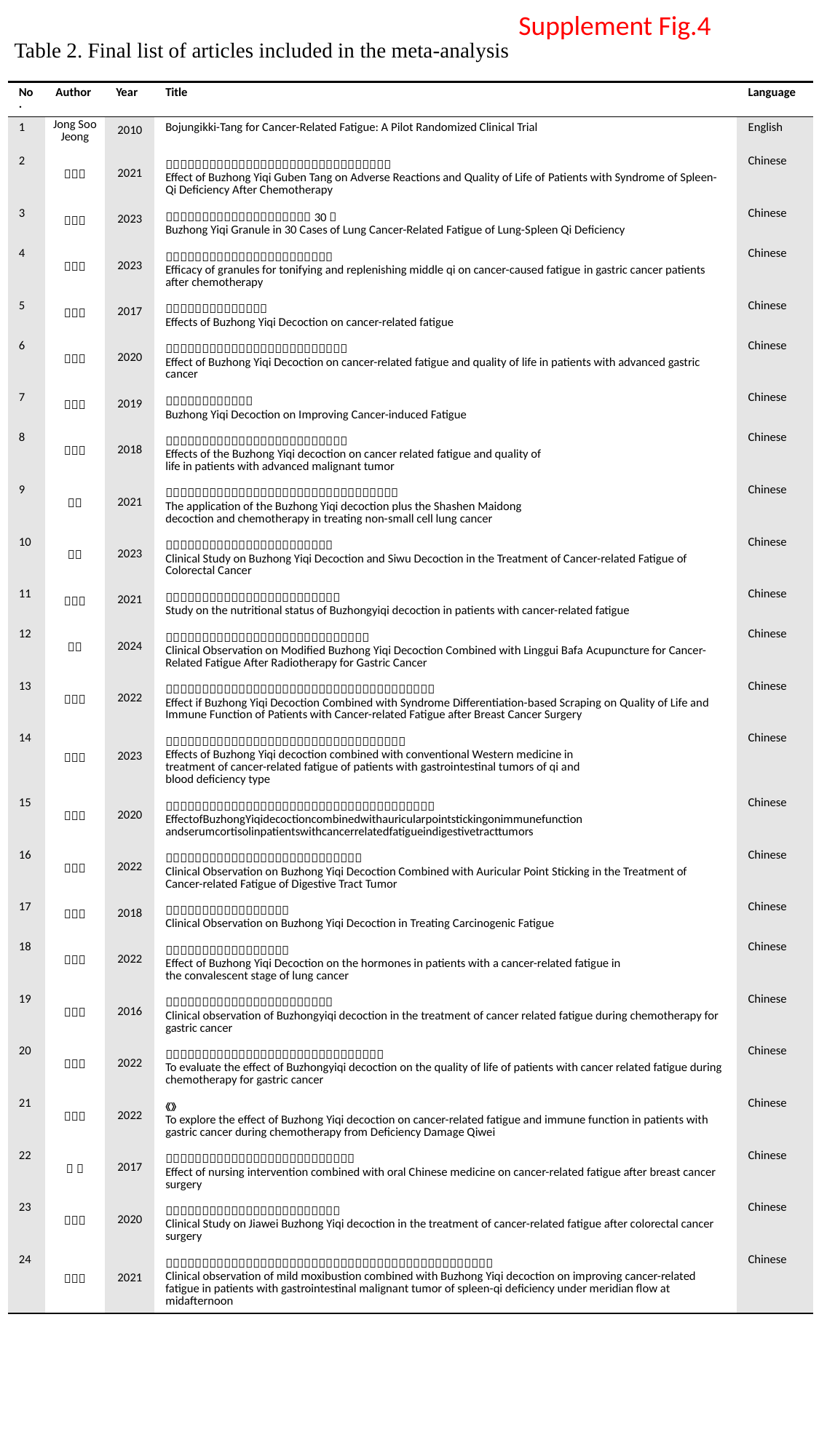

Supplement Fig.4
Table 2. Final list of articles included in the meta-analysis
| No. | Author | Year | Title | Language |
| --- | --- | --- | --- | --- |
| 1 | Jong Soo Jeong | 2010 | Bojungikki-Tang for Cancer-Related Fatigue: A Pilot Randomized Clinical Trial | English |
| 2 | 高可新 | 2021 | 补中益气固本汤对脾气虚证癌症患者化疗后不良反应及生活质量的影响 Effect of Buzhong Yiqi Guben Tang on Adverse Reactions and Quality of Life of Patients with Syndrome of Spleen-Qi Deficiency After Chemotherapy | Chinese |
| 3 | 梅莎莎 | 2023 | 补中益气颗粒治疗肺脾气虚型肺癌癌因性疲乏30例 Buzhong Yiqi Granule in 30 Cases of Lung Cancer-Related Fatigue of Lung-Spleen Qi Deficiency | Chinese |
| 4 | 苏羚子 | 2023 | 补中益气颗粒治疗胃癌化疗患者癌因性疲乏疗效观察 Efficacy of granules for tonifying and replenishing middle qi on cancer-caused fatigue in gastric cancer patients after chemotherapy | Chinese |
| 5 | 陈志成 | 2017 | 补中益气汤对癌因性疲乏的影响 Effects of Buzhong Yiqi Decoction on cancer-related fatigue | Chinese |
| 6 | 谢燕华 | 2020 | 补中益气汤对晚期胃癌患者癌因性疲乏及生存质量的影响 Effect of Buzhong Yiqi Decoction on cancer-related fatigue and quality of life in patients with advanced gastric cancer | Chinese |
| 7 | 于建华 | 2019 | 补中益气汤改善癌因性疲乏 Buzhong Yiqi Decoction on Improving Cancer-induced Fatigue | Chinese |
| 8 | 杨昌卫 | 2018 | 补中益气汤改善晚期恶性肿瘤患者癌因性疲乏的临床探讨 Effects of the Buzhong Yiqi decoction on cancer related fatigue and quality of life in patients with advanced malignant tumor | Chinese |
| 9 | 谭刚 | 2021 | 补中益气汤合沙参麦冬汤加减辅助化疗在非小细胞肺癌患者中的应用观察 The application of the Buzhong Yiqi decoction plus the Shashen Maidong decoction and chemotherapy in treating non-small cell lung cancer | Chinese |
| 10 | 王欣 | 2023 | 补中益气汤合四物汤治疗大肠癌癌因性疲乏临床观察 Clinical Study on Buzhong Yiqi Decoction and Siwu Decoction in the Treatment of Cancer-related Fatigue of Colorectal Cancer | Chinese |
| 11 | 段春燕 | 2021 | 补中益气汤加减方改善癌因性疲乏患者的营养状况研究 Study on the nutritional status of Buzhongyiqi decoction in patients with cancer-related fatigue | Chinese |
| 12 | 梁冰 | 2024 | 补中益气汤加减联合灵龟八法治疗胃癌放疗后癌性疲乏临床研究 Clinical Observation on Modified Buzhong Yiqi Decoction Combined with Linggui Bafa Acupuncture for Cancer-Related Fatigue After Radiotherapy for Gastric Cancer | Chinese |
| 13 | 张新庆 | 2022 | 补中益气汤联合辨证刮痧对乳腺癌术后癌性疲劳患者生活质量及免疫功能的改善作用 Effect if Buzhong Yiqi Decoction Combined with Syndrome Differentiation-based Scraping on Quality of Life and Immune Function of Patients with Cancer-related Fatigue after Breast Cancer Surgery | Chinese |
| 14 | 李欣欣 | 2023 | 补中益气汤联合常规西医治疗气血两虚型消化道肿瘤癌因性疲乏患者的效果 Effects of Buzhong Yiqi decoction combined with conventional Western medicine in treatment of cancer-related fatigue of patients with gastrointestinal tumors of qi and blood deficiency type | Chinese |
| 15 | 陈诗园 | 2020 | 补中益气汤联合耳穴贴压对消化道肿瘤癌因性疲乏患者免疫功能及血清皮质醇的影响 EffectofBuzhongYiqidecoctioncombinedwithauricularpointstickingonimmunefunction andserumcortisolinpatientswithcancerrelatedfatigueindigestivetracttumors | Chinese |
| 16 | 李恩强 | 2022 | 补中益气汤联合耳穴压贴治疗消化道肿瘤癌因性疲乏临床观察 Clinical Observation on Buzhong Yiqi Decoction Combined with Auricular Point Sticking in the Treatment of Cancer-related Fatigue of Digestive Tract Tumor | Chinese |
| 17 | 林振荣 | 2018 | 补中益气汤治疗癌因性疲乏的临床观察 Clinical Observation on Buzhong Yiqi Decoction in Treating Carcinogenic Fatigue | Chinese |
| 18 | 姜爱萍 | 2022 | 补中益气汤治疗肺癌康复期癌因性疲乏 Effect of Buzhong Yiqi Decoction on the hormones in patients with a cancer-related fatigue in the convalescent stage of lung cancer | Chinese |
| 19 | 朱国栋 | 2016 | 补中益气汤治疗胃癌化疗间期癌因性疲乏的临床观察 Clinical observation of Buzhongyiqi decoction in the treatment of cancer related fatigue during chemotherapy for gastric cancer | Chinese |
| 20 | 王国华 | 2022 | 补中益气汤治疗胃癌化疗间期癌因性疲乏对患者生活质量的改善评价 To evaluate the effect of Buzhongyiqi decoction on the quality of life of patients with cancer related fatigue during chemotherapy for gastric cancer | Chinese |
| 21 | 刘海洋 | 2022 | 从《虚损启微》中探析补中益气汤对胃癌化疗间期患者癌因性疲乏及免疫功能的影响 To explore the effect of Buzhong Yiqi decoction on cancer-related fatigue and immune function in patients with gastric cancer during chemotherapy from Deficiency Damage Qiwei | Chinese |
| 22 | 陈 宁 | 2017 | 护理干预联合中药内服治疗乳腺癌术后癌因性疲乏效果观察 Effect of nursing intervention combined with oral Chinese medicine on cancer-related fatigue after breast cancer surgery | Chinese |
| 23 | 宁博彪 | 2020 | 加味补中益气汤治疗大肠癌术后癌因性疲乏的临床研究 Clinical Study on Jiawei Buzhong Yiqi decoction in the treatment of cancer-related fatigue after colorectal cancer surgery | Chinese |
| 24 | 季尹霞 | 2021 | 子午流注下经络循行温和灸联合补中益气汤在改善脾气虚型胃肠道恶性肿瘤患者癌因性疲乏的临床观察 Clinical observation of mild moxibustion combined with Buzhong Yiqi decoction on improving cancer-related fatigue in patients with gastrointestinal malignant tumor of spleen-qi deficiency under meridian flow at midafternoon | Chinese |

## Slide 7
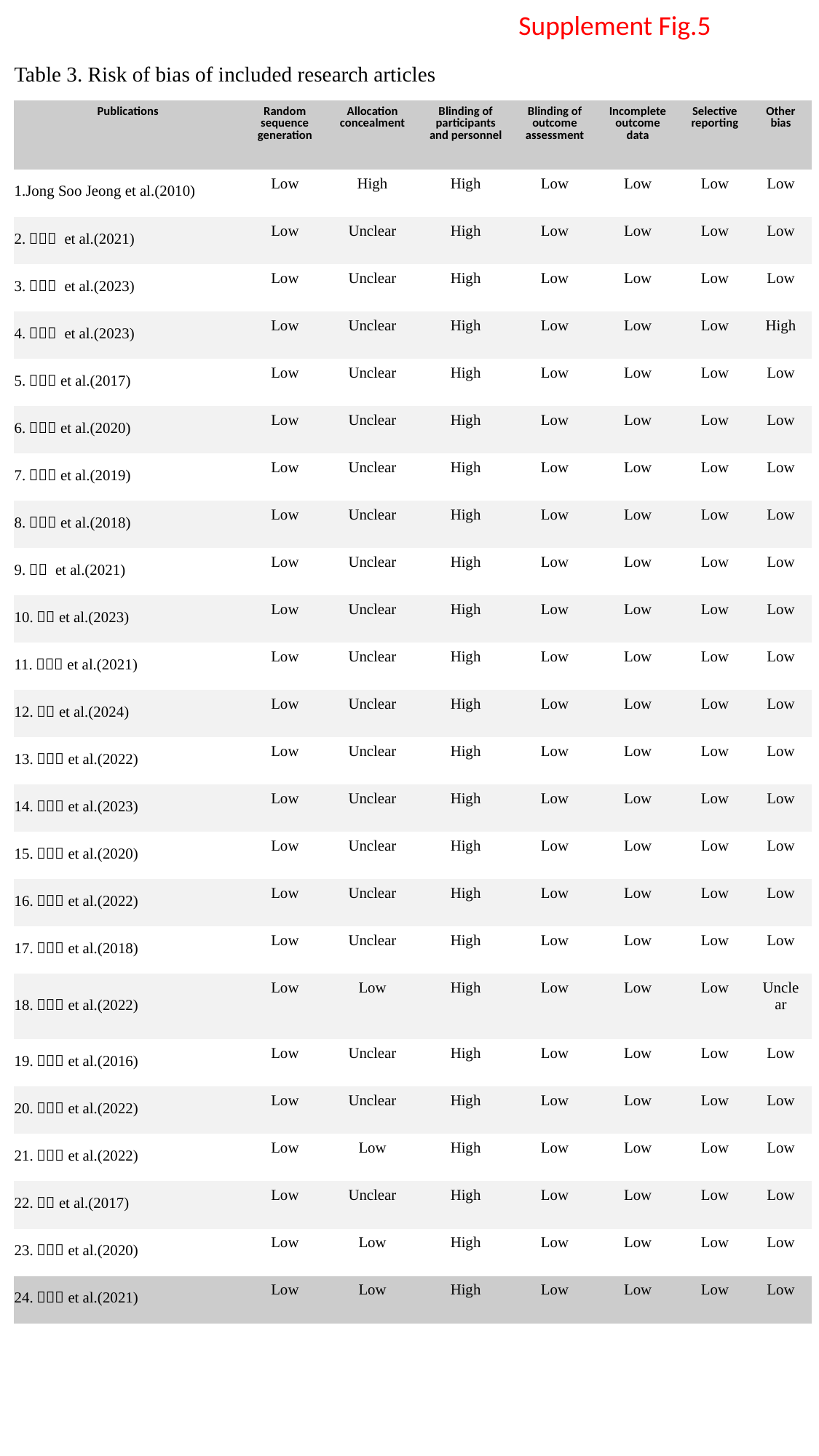

Supplement Fig.5
Table 3. Risk of bias of included research articles
| Publications | Random sequence generation | Allocation concealment | Blinding of participants and personnel | Blinding of outcome assessment | Incomplete outcome data | Selective reporting | Other bias |
| --- | --- | --- | --- | --- | --- | --- | --- |
| 1.Jong Soo Jeong et al.(2010) | Low | High | High | Low | Low | Low | Low |
| 2.高可新 et al.(2021) | Low | Unclear | High | Low | Low | Low | Low |
| 3.梅莎莎 et al.(2023) | Low | Unclear | High | Low | Low | Low | Low |
| 4.苏羚子 et al.(2023) | Low | Unclear | High | Low | Low | Low | High |
| 5.陈志成et al.(2017) | Low | Unclear | High | Low | Low | Low | Low |
| 6.谢燕华et al.(2020) | Low | Unclear | High | Low | Low | Low | Low |
| 7.于建华et al.(2019) | Low | Unclear | High | Low | Low | Low | Low |
| 8.杨昌卫et al.(2018) | Low | Unclear | High | Low | Low | Low | Low |
| 9.谭刚 et al.(2021) | Low | Unclear | High | Low | Low | Low | Low |
| 10.王欣et al.(2023) | Low | Unclear | High | Low | Low | Low | Low |
| 11.段春燕et al.(2021) | Low | Unclear | High | Low | Low | Low | Low |
| 12.梁冰et al.(2024) | Low | Unclear | High | Low | Low | Low | Low |
| 13.张新庆et al.(2022) | Low | Unclear | High | Low | Low | Low | Low |
| 14.李欣欣et al.(2023) | Low | Unclear | High | Low | Low | Low | Low |
| 15.陈诗园et al.(2020) | Low | Unclear | High | Low | Low | Low | Low |
| 16.李恩强et al.(2022) | Low | Unclear | High | Low | Low | Low | Low |
| 17.林振荣et al.(2018) | Low | Unclear | High | Low | Low | Low | Low |
| 18.姜爱萍et al.(2022) | Low | Low | High | Low | Low | Low | Unclear |
| 19.朱国栋et al.(2016) | Low | Unclear | High | Low | Low | Low | Low |
| 20.王国华et al.(2022) | Low | Unclear | High | Low | Low | Low | Low |
| 21.刘海洋et al.(2022) | Low | Low | High | Low | Low | Low | Low |
| 22.陈宁et al.(2017) | Low | Unclear | High | Low | Low | Low | Low |
| 23.宁博彪et al.(2020) | Low | Low | High | Low | Low | Low | Low |
| 24.季尹霞et al.(2021) | Low | Low | High | Low | Low | Low | Low |

## Slide 8
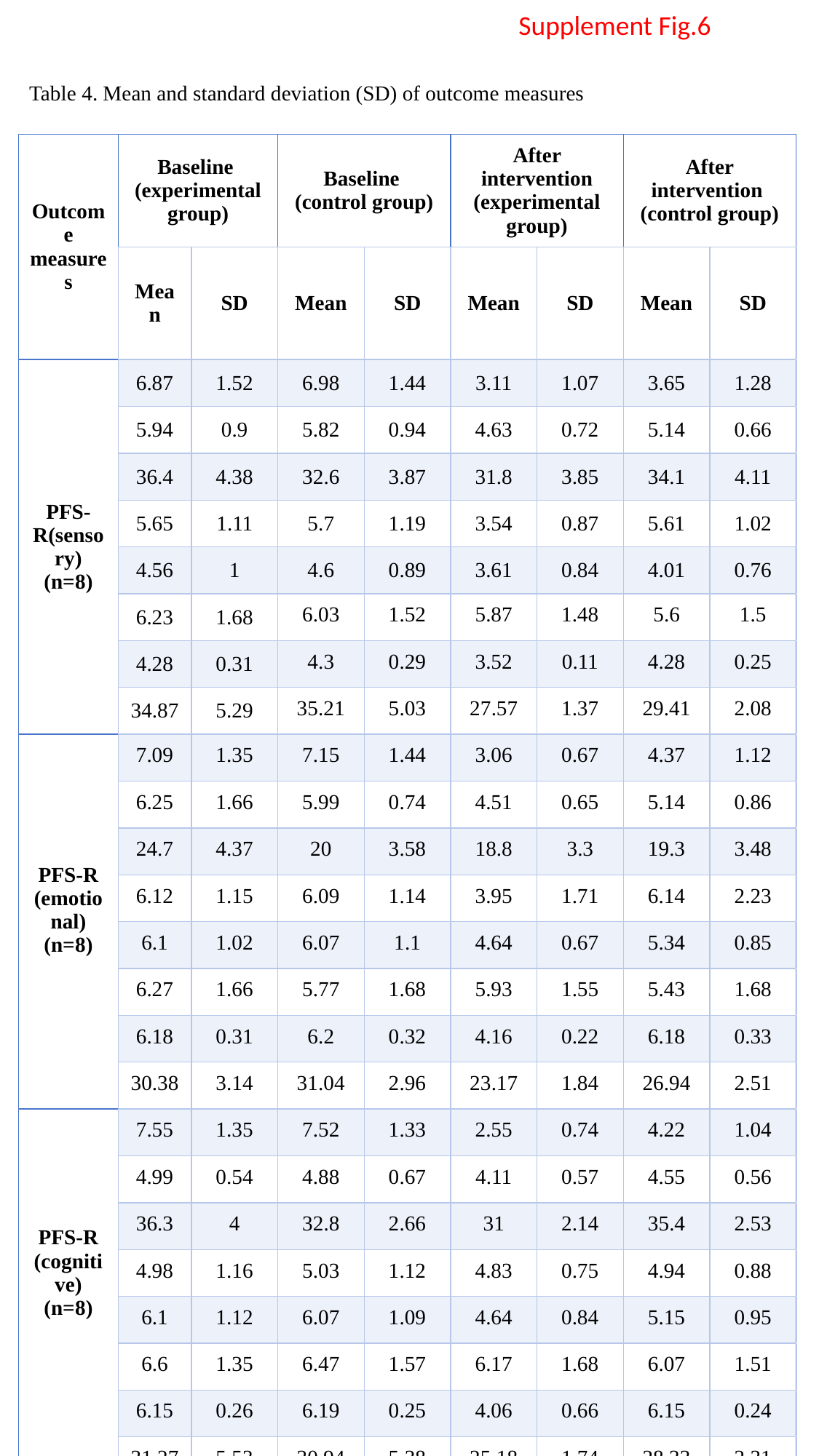

Supplement Fig.6
Table 4. Mean and standard deviation (SD) of outcome measures
| Outcome measures | Baseline (experimental group) | | Baseline (control group) | | After intervention (experimental group) | | After intervention (control group) | |
| --- | --- | --- | --- | --- | --- | --- | --- | --- |
| | Mean | SD | Mean | SD | Mean | SD | Mean | SD |
| PFS-R(sensory) (n=8) | 6.87 | 1.52 | 6.98 | 1.44 | 3.11 | 1.07 | 3.65 | 1.28 |
| | 5.94 | 0.9 | 5.82 | 0.94 | 4.63 | 0.72 | 5.14 | 0.66 |
| | 36.4 | 4.38 | 32.6 | 3.87 | 31.8 | 3.85 | 34.1 | 4.11 |
| | 5.65 | 1.11 | 5.7 | 1.19 | 3.54 | 0.87 | 5.61 | 1.02 |
| | 4.56 | 1 | 4.6 | 0.89 | 3.61 | 0.84 | 4.01 | 0.76 |
| | 6.23 | 1.68 | 6.03 | 1.52 | 5.87 | 1.48 | 5.6 | 1.5 |
| | 4.28 | 0.31 | 4.3 | 0.29 | 3.52 | 0.11 | 4.28 | 0.25 |
| | 34.87 | 5.29 | 35.21 | 5.03 | 27.57 | 1.37 | 29.41 | 2.08 |
| PFS-R (emotional) (n=8) | 7.09 | 1.35 | 7.15 | 1.44 | 3.06 | 0.67 | 4.37 | 1.12 |
| | 6.25 | 1.66 | 5.99 | 0.74 | 4.51 | 0.65 | 5.14 | 0.86 |
| | 24.7 | 4.37 | 20 | 3.58 | 18.8 | 3.3 | 19.3 | 3.48 |
| | 6.12 | 1.15 | 6.09 | 1.14 | 3.95 | 1.71 | 6.14 | 2.23 |
| | 6.1 | 1.02 | 6.07 | 1.1 | 4.64 | 0.67 | 5.34 | 0.85 |
| | 6.27 | 1.66 | 5.77 | 1.68 | 5.93 | 1.55 | 5.43 | 1.68 |
| | 6.18 | 0.31 | 6.2 | 0.32 | 4.16 | 0.22 | 6.18 | 0.33 |
| | 30.38 | 3.14 | 31.04 | 2.96 | 23.17 | 1.84 | 26.94 | 2.51 |
| PFS-R (cognitive) (n=8) | 7.55 | 1.35 | 7.52 | 1.33 | 2.55 | 0.74 | 4.22 | 1.04 |
| | 4.99 | 0.54 | 4.88 | 0.67 | 4.11 | 0.57 | 4.55 | 0.56 |
| | 36.3 | 4 | 32.8 | 2.66 | 31 | 2.14 | 35.4 | 2.53 |
| | 4.98 | 1.16 | 5.03 | 1.12 | 4.83 | 0.75 | 4.94 | 0.88 |
| | 6.1 | 1.12 | 6.07 | 1.09 | 4.64 | 0.84 | 5.15 | 0.95 |
| | 6.6 | 1.35 | 6.47 | 1.57 | 6.17 | 1.68 | 6.07 | 1.51 |
| | 6.15 | 0.26 | 6.19 | 0.25 | 4.06 | 0.66 | 6.15 | 0.24 |
| | 31.27 | 5.53 | 30.94 | 5.38 | 25.18 | 1.74 | 28.33 | 2.31 |

## Slide 9
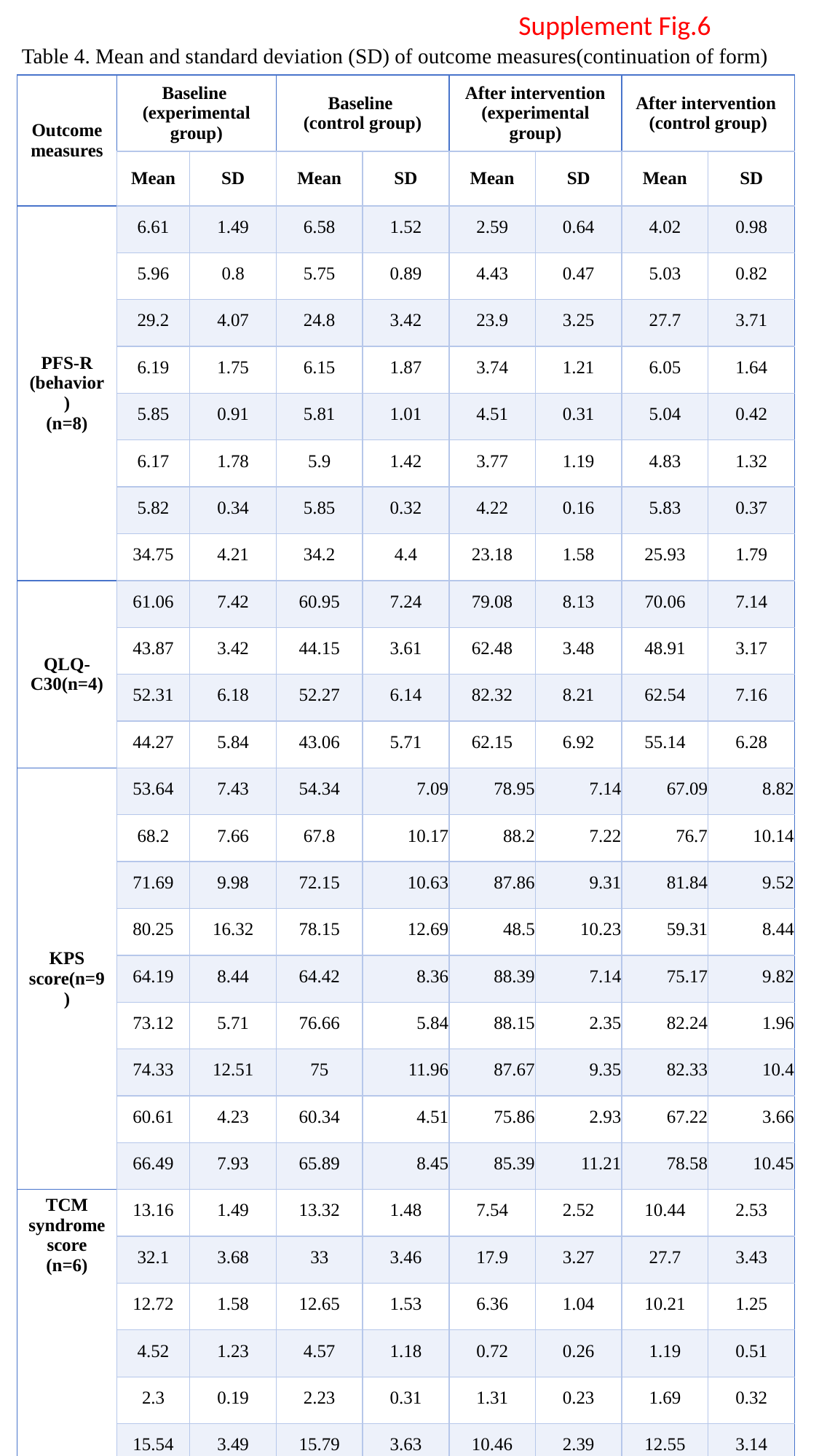

Supplement Fig.6
Table 4. Mean and standard deviation (SD) of outcome measures(continuation of form)
| Outcome measures | Baseline (experimental group) | | Baseline (control group) | | After intervention (experimental group) | | After intervention (control group) | |
| --- | --- | --- | --- | --- | --- | --- | --- | --- |
| | Mean | SD | Mean | SD | Mean | SD | Mean | SD |
| PFS-R (behavior) (n=8) | 6.61 | 1.49 | 6.58 | 1.52 | 2.59 | 0.64 | 4.02 | 0.98 |
| | 5.96 | 0.8 | 5.75 | 0.89 | 4.43 | 0.47 | 5.03 | 0.82 |
| | 29.2 | 4.07 | 24.8 | 3.42 | 23.9 | 3.25 | 27.7 | 3.71 |
| | 6.19 | 1.75 | 6.15 | 1.87 | 3.74 | 1.21 | 6.05 | 1.64 |
| | 5.85 | 0.91 | 5.81 | 1.01 | 4.51 | 0.31 | 5.04 | 0.42 |
| | 6.17 | 1.78 | 5.9 | 1.42 | 3.77 | 1.19 | 4.83 | 1.32 |
| | 5.82 | 0.34 | 5.85 | 0.32 | 4.22 | 0.16 | 5.83 | 0.37 |
| | 34.75 | 4.21 | 34.2 | 4.4 | 23.18 | 1.58 | 25.93 | 1.79 |
| QLQ-C30(n=4) | 61.06 | 7.42 | 60.95 | 7.24 | 79.08 | 8.13 | 70.06 | 7.14 |
| | 43.87 | 3.42 | 44.15 | 3.61 | 62.48 | 3.48 | 48.91 | 3.17 |
| | 52.31 | 6.18 | 52.27 | 6.14 | 82.32 | 8.21 | 62.54 | 7.16 |
| | 44.27 | 5.84 | 43.06 | 5.71 | 62.15 | 6.92 | 55.14 | 6.28 |
| KPS score(n=9) | 53.64 | 7.43 | 54.34 | 7.09 | 78.95 | 7.14 | 67.09 | 8.82 |
| | 68.2 | 7.66 | 67.8 | 10.17 | 88.2 | 7.22 | 76.7 | 10.14 |
| | 71.69 | 9.98 | 72.15 | 10.63 | 87.86 | 9.31 | 81.84 | 9.52 |
| | 80.25 | 16.32 | 78.15 | 12.69 | 48.5 | 10.23 | 59.31 | 8.44 |
| | 64.19 | 8.44 | 64.42 | 8.36 | 88.39 | 7.14 | 75.17 | 9.82 |
| | 73.12 | 5.71 | 76.66 | 5.84 | 88.15 | 2.35 | 82.24 | 1.96 |
| | 74.33 | 12.51 | 75 | 11.96 | 87.67 | 9.35 | 82.33 | 10.4 |
| | 60.61 | 4.23 | 60.34 | 4.51 | 75.86 | 2.93 | 67.22 | 3.66 |
| | 66.49 | 7.93 | 65.89 | 8.45 | 85.39 | 11.21 | 78.58 | 10.45 |
| TCM syndrome score (n=6) | 13.16 | 1.49 | 13.32 | 1.48 | 7.54 | 2.52 | 10.44 | 2.53 |
| | 32.1 | 3.68 | 33 | 3.46 | 17.9 | 3.27 | 27.7 | 3.43 |
| | 12.72 | 1.58 | 12.65 | 1.53 | 6.36 | 1.04 | 10.21 | 1.25 |
| | 4.52 | 1.23 | 4.57 | 1.18 | 0.72 | 0.26 | 1.19 | 0.51 |
| | 2.3 | 0.19 | 2.23 | 0.31 | 1.31 | 0.23 | 1.69 | 0.32 |
| | 15.54 | 3.49 | 15.79 | 3.63 | 10.46 | 2.39 | 12.55 | 3.14 |

## Slide 10
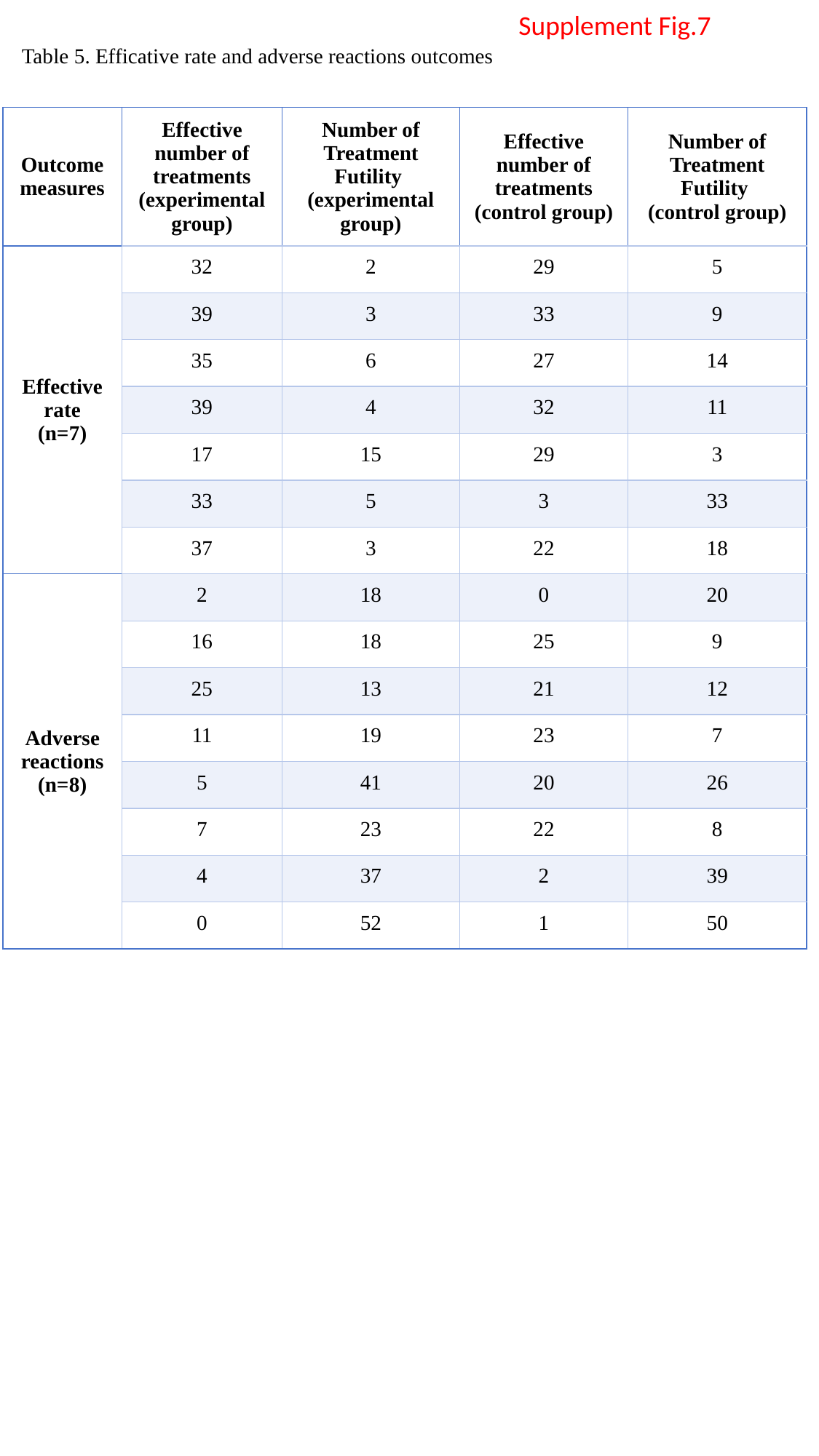

Supplement Fig.7
Table 5. Efficative rate and adverse reactions outcomes
| Outcome measures | Effective number of treatments (experimental group) | Number of Treatment Futility (experimental group) | Effective number of treatments (control group) | Number of Treatment Futility (control group) |
| --- | --- | --- | --- | --- |
| Effective rate (n=7) | 32 | 2 | 29 | 5 |
| | 39 | 3 | 33 | 9 |
| | 35 | 6 | 27 | 14 |
| | 39 | 4 | 32 | 11 |
| | 17 | 15 | 29 | 3 |
| | 33 | 5 | 3 | 33 |
| | 37 | 3 | 22 | 18 |
| Adverse reactions (n=8) | 2 | 18 | 0 | 20 |
| | 16 | 18 | 25 | 9 |
| | 25 | 13 | 21 | 12 |
| | 11 | 19 | 23 | 7 |
| | 5 | 41 | 20 | 26 |
| | 7 | 23 | 22 | 8 |
| | 4 | 37 | 2 | 39 |
| | 0 | 52 | 1 | 50 |

## Slide 11
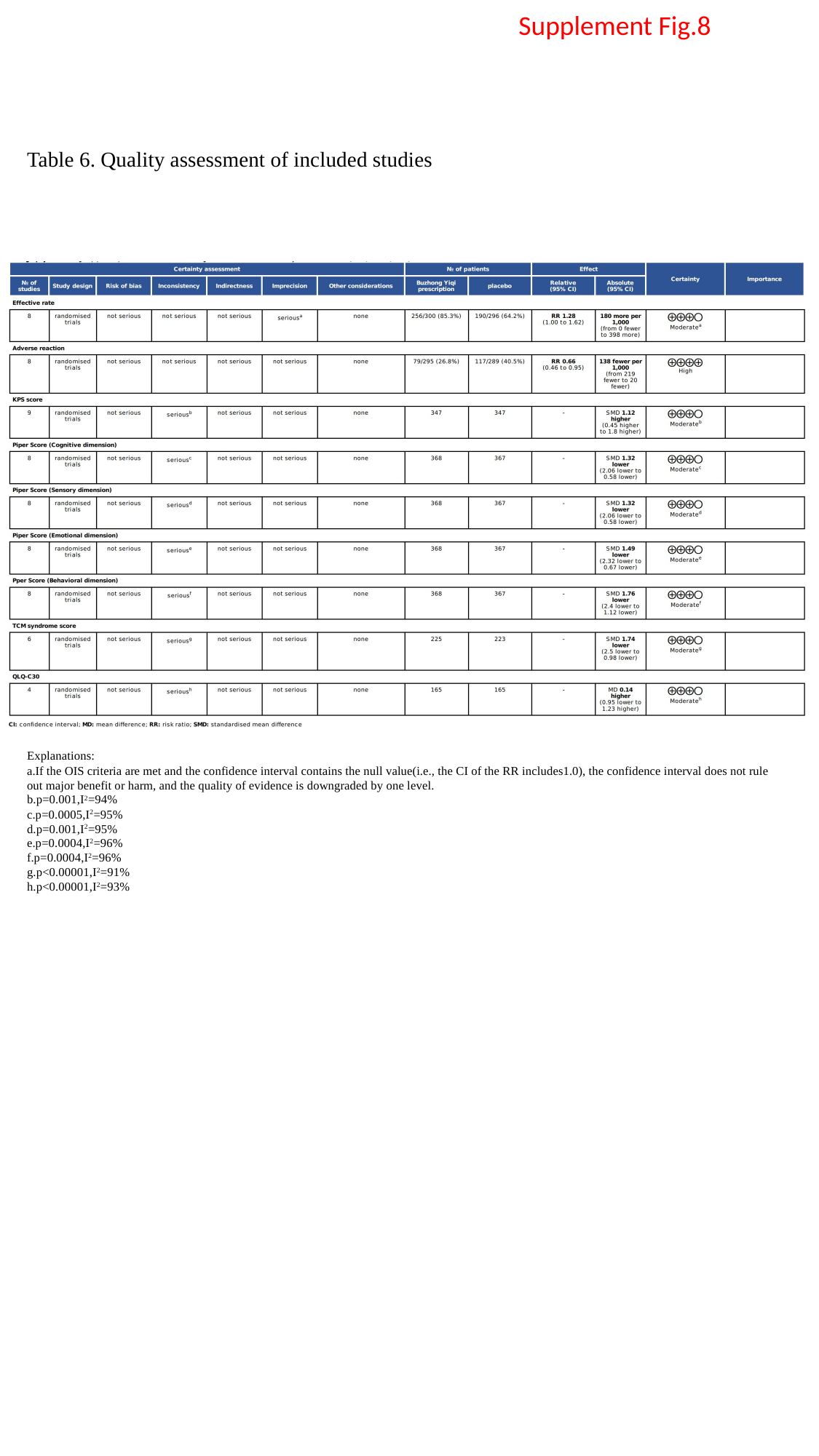

Supplement Fig.8
Table 6. Quality assessment of included studies
Explanations:
a.If the OIS criteria are met and the confidence interval contains the null value(i.e., the CI of the RR includes1.0), the confidence interval does not rule out major benefit or harm, and the quality of evidence is downgraded by one level.
b.p=0.001,I2=94%
c.p=0.0005,I2=95%
d.p=0.001,I2=95%
e.p=0.0004,I2=96%
f.p=0.0004,I2=96%
g.p<0.00001,I2=91%
h.p<0.00001,I2=93%
